# Supplementary material for: Qualitative exploration of the intersection between social influences and cultural norms in relation to the development of alcohol use behaviour during adolescence
Source: BMJ Open. 2020 Mar 16;10(3):e030556. doi: 10.1136/bmjopen-2019-030556 (PMC7076248; doi:10.1136/bmjopen-2019-030556)
Supplement: Supplementary data [file bmjopen-2019-030556supp001.pdf]

Supplementary Material: Additional quotes

S1. Normalisation of drinking and the wider alcohol culture

1. *Yes but also like in films and like series, and social media, parties just look like crazy cus everyone's like getting drunk, so people who kind of their aim is, I wanna be cool, I wanna have lots of friends, and I wanna be the popular one. They think, the only way to do that is to go to the parties, cus that's what all the films say. (ID 13b, F, non-drinker)*

2. *I didn't really know that what it can actually do to you. I just thought oh it makes you happy and that's it, that's all you really hear about. (ID 21, F, drinker)*

3. *RES: … I think drinking [sic: in adults] is fine because like obviously people really enjoy like wine and stuff, um, but I think it's bad when you're like addicted or like drinking more than is necessary. Yeah.*
   INT: And how much would that be do you reckon, the drinking more than you need to?
   RES: Um, like, I dunno, if someone was drinking like a bottle of wine a day that's like… that's just like someone who's like… it's like kind of like a normal amount to drink but it's a lot. But if someone's actually drunk throughout the day that's terrible I think. Yeah. (ID 17, F, drinker)

4. *Well there's a point where it's like, not fun anymore and also, it's just not cool anymore. It can be seen as like, cool having a few cans, getting drunk but then like after that it's just silly. And people will like, ignore you, in that situation (ID 13b, F, non-drinker)*

MacArthur GJ, *et al. BMJ Open* 2020; 10:e030556. doi: 10.1136/bmjopen-2019-030556

5. *INT: Yeah, and what might be some of the bad things that you mention there?*

RES: Getting addicted or feeling sick the next day. You can do bad things when you're drunk.

INT: Bad things?

RES: Well you don't really know what you're doing. (ID 40, M, light drinker)

6. *INT: And when you say kind of light drinking can I ask a bit what that would be?*

RES: Maybe slightly tipsy but then like to the point where you can't walk is… there's n… there's no point.

INT: Yeah. Okay. So, up to that point it's…?

RES: Well, slightly tipsy, [inaudible] but then anything more is pretty pointless but… yeah.

INT: Yeah. And pointless because?

RES: It just… it's sort of taking the fun out of it and it's just… you're just being stupid and not controlling at all.

INT: Hmm. And what sort of things might happen at that point then?

RES: They'd hurt themselves.

INT: Okay. Yeah.

RES: Er, could hurt others maybe. Yeah. (ID 22, M, light drinker)

MacArthur GJ, *et al. BMJ Open* 2020; 10:e030556. doi: 10.1136/bmjopen-2019-030556

S2. Motivations to drink and the initiation of alcohol use

1. *.. you could forget your troubles maybe and just live in the moment and just go, 'I'll deal with the problems later. I'll have fun now'. (ID 22, M, light drinker)*

2. *I think it's a good social thing like it makes it easier for you to talk to people, you know, and you can - you don't have to worry about like making a fool of yourself because you have an excuse and stuff. (ID 18, F, drinker)*

S3: Young people's drinking and party culture

1. *People are saying oh its so, it will be so dead like without them drinking, like it will just be so boring and stuff, but they say like oh its better when we are drinking because it's more fun and like everybody is falling and everybody is like laughing. (ID 6, F, non-drinker)*

2. *Yeah, some people like – some people might usually go into like say the party and stuff and just say like "Oh I wanna get smashed" or something like that. (ID 36, M, drinker)*

3. *It's just mainly like parties, I guess, like people just think, it's a party, you have to drink, basically. (35b, F, non-drinker)*

4. *I'm not like – people often call you, um, a lightweight when you drink and get drunk, and it's like that's an offensive term. It's quite embarrassing if you get called that, but yeah, I think there's a lot of stigma towards that. (ID 32, F, drinker)*

5. *RES: I could be their friend and then they could do something stupid and I'd have to look after them or go and try and fix it, um, yeah.*
   INT: And how would that make you feel doing that?
   RES: Quite annoyed because they got themselves like this. (ID 22, M, light drinker)

MacArthur GJ, *et al. BMJ Open* 2020; 10:e030556. doi: 10.1136/bmjopen-2019-030556

S4. Social influences on adolescent alcohol use

4.1. Peer influence

1. *INT: And what do you think might have changed those people's opinion, how you said some people didn't and now do?*

   RES: Probably because they've seen the first people do it and once somebody…

   INT: Hmm.

   RES: … one person starts doing it and then the next person then I think eventually it becomes like, everyone just wants to try it and then if they like it then they'll do it again and again. (ID 34, F, moderate drinker)

2. *Like one party we were at there's a group of people and they were talking about revision and so you just like walk away from them, and you're like 'oh, they're such like a buzz-kill' because it's not… they don't have to be drunk but they're just like changing… they're like shifting the… like the, um, like the vibe of the party to more like, I dunno, not as funny. (ID 17, F, drinker)*

3. *INT: What do you think are the main reasons people might drink?*

   RES: Um, just they're eager to drink, and their friends are drinking. They will just drink. Like, it's... I mean, sometimes it's not even peer pressure; it's just seeing other people doing it, and you just want to do it as well, just to fit in. (ID 16, M, non-drinker)

4. *I know definitely that I felt a bit like not left out but like, not part of it, that's why I didn't enjoy it. It was really boring cus like I didn't wanna talk to anyone because*

they were all just like really drunk. So I just stayed like half an hour. (ID 13a, F, non-drinker)

5. *RES: Maybe like if you're invited to a party and everyone else there. You don't really wanna be the one that…the only one not.*

   INT: Um. How would it feel if you were the only person who wasn't do you think?

   RES: Embarrassed maybe.

   INT: Um and why do you think that is?

   RES: Um 'cause you don't feel like you fit in the group. (ID 40, M, light drinker)

6. *But I think it's more like yeah you don't want to be sober at a party if you don't want to be the person to look after all your friends.* (ID 21, F, drinker)

7. *Because they see people when they're drunk and they'll think well that… that may… may… you may think is fun but it really doesn't look that fun cos you're jumping about and jumping on your head and stuff like that. What's the point? (ID 22, M, light drinker)*

8. *RES 1: .. in my friendship group I'm kind of like the mum like I'm always like.*

   RES 2: Me too.

   RES 1: I'm always like just making sure everyone's ok.

   RES 2: Checking everyone's all right. (ID 31a F; 31b, F, moderate drinkers)

9. *I have had the invites from my friends but I've chosen not to go because I find that sport is more important to me, than just going drinking. (ID 37, M, non-drinker)*

4.2. The influence of social media

1. *They kind of make you feel like you want to drink more cus it's like they're having... When you look on Snapchat, most of the time, it just seems like you can see, like, the good things. Cos people filming it are like oh such good fun. They won't put on stuff that's like really bad. So it makes it; like I guess it gives the alcohol like a more positive view (ID 8b, F, non-drinker)*

2. *RES: I think whenever people are having like a night, like party then there's always like drinks on their Snapchat which just shows that they're having fun and they're drinking…*

   INT: Mm.

   RES: So I wanna do that. And go to more things like that. (ID 26, F, drinker)

S5. The pressurised environment

1. *INT: What kind of things would people say? What sort of um, type of thing would they be?*

   RES: Just like – like calling them scared and stuff for not doing it and stuff like that.

   INT: Okay. Yeah. Yeah.

   RES: Like, are you too scared to do this? But like other words. (ID 33, M, drinker)

2. *.. a lot of people drink to impress their friends so say like one friends like oh yeah I can drink this much and then the others like well I can drink this much and it's almost like a sly competition and then that's when it gets bad and out of hand like I've seen that from friends and then they've lost friends like because of it. (ID 31a, F, moderate drinker)*

3. *RES: I think it's harder because what people want other people's – they want other people to think about them like in a more positive way like…*

   INT: Hmm.

   RES: … they don't wanna be thought of this person who's like not fun and doesn't want to do all the stuff… (ID 33, M, drinker)

4. *RES3: Some people think you have to, just like peer pressure, I guess, if you're at a party or something [laughs].*

   RES2: Mm.

   INT: What does that kind of feel like? What happens, when you say, 'peer pressure', what's...?

   RES1: Forced into doing it, really. (ID 35a (M), b (F), c (F), non-drinkers)

5. *RES: I think they always teach like I think they always teach peer pressure wrong.*

   INT: Right. So what do they say?

   RES: I mean they - well they always just say like if someone asks you to do something that you don't want to do just say no. But it-it's not really that. I think it's more that people are doing things and if you are not part of it – well you're not – like if you're not doing it as well you're not part of it... (ID 7, F, non-drinker)

6. *RES: I don't think so. I think I haven't really ever experienced any, like, peer pressure. Um, but I still think there is quite a lot of pressure to drink, um, whether or not anyone means to pressurise you. (ID 32, F, drinker)*

Abbreviations:

INT: Interviewer

RES: respondent

MacArthur GJ, *et al. BMJ Open* 2020; 10:e030556. doi: 10.1136/bmjopen-2019-030556
