# Supplementary material for: Qualitative exploration of the intersection between social influences and cultural norms in relation to the development of alcohol use behaviour during adolescence
Source: BMJ Open. 2020 Mar 16;10(3):e030556. doi: 10.1136/bmjopen-2019-030556 (PMC7076248; doi:10.1136/bmjopen-2019-030556)
Supplement: Author's manuscript [file bmjopen-2019-030556.draft_revisions.pdf]

BMJ Open is committed to open peer review. As part of this commitment we make the peer review history of every article we publish publicly available.

When an article is published we post the peer reviewers' comments and the authors' responses online. We also post the versions of the paper that were used during peer review. These are the versions that the peer review comments apply to.

The versions of the paper that follow are the versions that were submitted during the peer review process. They are not the versions of record or the final published versions. They should not be cited or distributed as the published version of this manuscript.

BMJ Open is an open access journal and the full, final, typeset and author-corrected version of record of the manuscript is available on our site with no access controls, subscription charges or pay-per-view fees (http://bmjopen.bmj.com).

If you have any questions on BMJ Open's open peer review process please email info.bmjopen@bmj.com

BMJ Open

A qualitative exploration of the intersection between social influences and cultural norms in relation to the development of alcohol use behaviour during adolescence.

Journal:	*BMJ Open*
Manuscript ID	bmjopen-2019-030556
Article Type:	Research
Date Submitted by the Author:	19-Mar-2019
Complete List of Authors:	MacArthur, George; Bristol University, Social and Community Medicine<br>Hickman, M; Bristol<br>Campbell, Rona; University of Bristol, School of Social and Community Medicine
Keywords:	QUALITATIVE RESEARCH, PREVENTIVE MEDICINE, PUBLIC HEALTH

SCHOLARONE™
Manuscripts

I, the Submitting Author has the right to grant and does grant on behalf of all authors of the Work (as defined in the below author licence), an exclusive licence and/or a non-exclusive licence for contributions from authors who are: i) UK Crown employees; ii) where BMJ has agreed a CC-BY licence shall apply, and/or iii) in accordance with the terms applicable for US Federal Government officers or employees acting as part of their official duties; on a worldwide, perpetual, irrevocable, royalty-free basis to BMJ Publishing Group Ltd ("BMJ") its licensees and where the relevant Journal is co-owned by BMJ to the co-owners of the Journal, to publish the Work in this journal and any other BMJ products and to exploit all rights, as set out in our licence.

The Submitting Author accepts and understands that any supply made under these terms is made by BMJ to the Submitting Author unless you are acting as an employee on behalf of your employer or a postgraduate student of an affiliated institution which is paying any applicable article publishing charge ("APC") for Open Access articles. Where the Submitting Author wishes to make the Work available on an Open Access basis (and intends to pay the relevant APC), the terms of reuse of such Open Access shall be governed by a Creative Commons licence – details of these licences and which Creative Commons licence will apply to this Work are set out in our licence referred to above.

Other than as permitted in any relevant BMJ Author's Self Archiving Policies, I confirm this Work has not been accepted for publication elsewhere, is not being considered for publication elsewhere and does not duplicate material already published. I confirm all authors consent to publication of this Work and authorise the granting of this licence.

For peer review only

A qualitative exploration of the intersection between social influences and cultural norms in relation to the development of alcohol use behaviour during adolescence.

MacArthur GJ[1], Hickman M[1], Campbell R[1]

Running title: Adolescent alcohol use: norms and influences.

[1] Population Health Sciences, Bristol Medical School, University of Bristol, Canynge Hall, 39 Whatley Road, Bristol BS8 2PS, UK.

MacArthur GJ PhD, NIHR postdoctoral research fellow, Department of Population Health Sciences, University of Bristol

Hickman M PhD, Professor of Epidemiology and Public Health, Department of Population Health Sciences, University of Bristol

Campbell R PhD, Professor of Public Health, Department of Population Health Sciences, University of Bristol

Corresponding author:

Address: Population Health Sciences, Bristol Medical School, University of Bristol, Canynge Hall, 39 Whatley Road, Bristol BS8 2PS, UK.

Email: georgie.macarthur@bristol.ac.uk

Tel: +44 (0) 117 928 7295

For peer review only

For peer review only - http://bmjopen.bmj.com/site/about/guidelines.xhtml

Abstract

Objectives

Few contemporary studies have examined peer and social drivers of alcohol use during mid-adolescence. We sought to explore young people's perspectives on socio-cultural influences relating to alcohol use behaviour during this period.

Design

Qualitative research study

Methods

Semi-structured one-to one (n=25), paired (n=3) or triad (n=1) interviews and one focus group (n=6) were conducted with thirty young people aged 14-15 (13 males, 17 females) recruited from four schools, and twelve participants (aged 14-18, 8 males, 4 females) recruited from two youth groups. Interviews were audio-recorded, transcribed verbatim and analysed thematically using NVivo 10.

Results

Alcohol consumption was associated with being cool, mature and popular, while enabling escape from reality and boosting confidence and enjoyment. Positive expectancies, alongside opportunity, contributed to motivating initiation, but social influences were paramount, with participants describing a need to 'fit in' with friends to avoid social exclusion. Such influences positioned drinking at parties as a normative social practice, providing opportunities for social learning and the strengthening of peer norms. Social media weaved into young people's lives positive alcohol-associated depictions of social status, enjoyment and maturity. This intersection of influences and norms generated a pressurised environment and a sense of unease around resisting pressures, which could elicit stigmatising insults.

Discussion and Conclusions

Cultural norms, social influences and social media intersect to create a pressurised environment around alcohol use during mid-adolescence, driving the escalation of excessive consumption. New

For peer review only - http://bmjopen.bmj.com/site/about/guidelines.xhtml

interventions need to address normative influences to enable the prevention of excessive alcohol use during adolescence.

Strengths and Limitations of this study

1. Strengths of the study include the gender-balanced sample of young people from both schools and youth groups, including those who used alcohol and those who abstained.
2. One-to-one interviews were conducted, which provided space for young people to share their views around drinking, without concerns around disagreement or judgement from peers or friends.
3. Limitations of this study include the lack of triangulation of data, and participants were recruited from urban and suburban settings only, meaning that our findings cannot necessarily be generalised to different geographical or demographic contexts.

Funding statement

GJM was supported by an NIHR post-doctoral fellowship award (PDF-2013-06-026). This report is independent research supported by the National Institute for Health Research (Post-Doctoral Fellowship, PDF-2013-06-026). The views expressed in this publication are those of the author(s) and not necessarily those of the NHS, the National Institute for Health Research or the Department of Health.

The work was undertaken with the support of The Centre for the Development and Evaluation of Complex Interventions for Public Health Improvement (DECIPHer), a UKCRC Public Health Research Centre of Excellence. Joint funding (MR/KO232331/1) from the British Heart Foundation, Cancer Research UK, Economic and Social Research Council, Medical Research Council, the Welsh Government and the Wellcome Trust, under the auspices of the UK Clinical Research Collaboration, is gratefully acknowledged.

RC is supported by the University of Bristol.

For peer review only - http://bmjopen.bmj.com/site/about/guidelines.xhtml

Competing interests

GJM declares no competing interests.

RC is a scientific advisor to DECIPHer Impact a not-for-profit company wholly owned by the Universities of Cardiff and Bristol which licences, quality-assures and supports the delivery of evidence-based public health promotion interventions. RC receives payment for this work.

MH has received payment for unrelated activity from Gilead Sciences Inc, Bristol Myers-Squibb and Jannsen, UK.

Contribution of authors:

GJM was responsible for recruitment, acquisition, analysis and interpretation of data and wrote the first draft of the manuscript. MH contributed to interpretation of data and finalisation of the manuscript. RC was responsible for conceptualisation of the study and contributed to interpretation of data and finalisation of the manuscript.

Keywords:

Alcohol drinking, qualitative, adolescent, social norms, peers

For peer review only

Introduction

Although downward trends in alcohol use have been reported in the US and Europe,(1, 2) the reasons for this decline are as yet unclear, and while evidence is scant, studies indicate that the decline may not be uniform across all socio-demographic or levels of alcohol consumption.(3) Despite declines in the UK, recent figures show that 24% of 15-year olds in the UK have had a drink in the last week,(4) and by the age of 17, half of girls, and nearly two-thirds of boys, report drinking every week.(5) Adolescent drinking in the UK therefore remains a public health concern. Guidance from the Chief Medical Officer for England states that an alcohol-free childhood is the healthiest and best option, and if children do drink alcohol, it should not be until at least the age of 15 years; while if those aged 15-17 do drink, they should do so infrequently and on no more than one day per week.(6)

The critical role of both peer and parental influences in relation to adolescent alcohol consumption is well-documented. Quantitative and qualitative studies have highlighted the impact of factors including parental monitoring, attitudes, communication and parental provision of alcohol via direct and indirect mechanisms,(7-12) while peer influences, norms, peer selection and the social context of drinking, together generate peer group-based behavioural drivers of alcohol consumption.(13-19)

Recent qualitative studies also highlight how the increasing engagement with online media and social networking sites (SNS) has enabled the sharing of stories and photographs, and managed displays of drinking and drinking behaviours, creating what have been termed as 'intoxigenic social identities' and 'intoxigenic digital spaces'.(20) Such spaces provide new platforms and opportunities for the positive promotion of alcohol consumption, creating an arena in which the display of identity, personality and popularity is carefully managed, and such networks therefore assist with the positioning of alcohol as central to social worlds.(21-23) Evidence also indicates an association

For peer review only - http://bmjopen.bmj.com/site/about/guidelines.xhtml

between exposure to friends' alcohol related content on SNS and alcohol use and later heavy episodic drinking, via the development of favourable peer injunctive norms.(24)

While qualitative studies have reported the effect of peer, parental and wider cultural influences on young people's drinking, interaction and engagement with SNS, and the integral nature of the social context,(13-15, 22, 25-27) few recent qualitative studies have involved an in-depth examination of social and cultural influences on the initiation and escalation of alcohol use during early- to mid-adolescence in England, and the interaction between such behavioural influences. We sought to obtain data on such influences that would be applicable to the national context to inform intervention development. In addition, since little data are available to help to explain downward trends in alcohol use among young people in England,(2, 3, 28) it also remains important to gain a contemporary understanding of the impact of peers, social influences and cultural factors to offer insights around factors that might affect such trends.

In this paper, we report findings of a qualitative study that aimed to explore the social, cultural and behavioural drivers of alcohol consumption and the nature of drinking culture in mid-adolescence. Our exploration of the social worlds and drinking cultures of younger adolescents, and influences on drinking practice, aims to inform the development of interventions to prevent excessive alcohol consumption and related harm.

For peer review only - http://bmjopen.bmj.com/site/about/guidelines.xhtml

Methods

While the aim of the study was to investigate the role of friendships in relation to drinking behaviour, we report here our findings regarding a wider range of determinants of behaviour, reflecting the topics pertinent to young people that emerged in the interviews.

Sampling and Recruitment

Our study sample included thirty young people aged 14-15 years who were recruited from four secondary schools in the South West of England. No participants dropped out of the study. The schools were in urban (n=3) and suburban/rural (n=1) areas and were located in areas with diverse levels of deprivation as reflected by the index of multiple deprivation score for the postcode. Students were randomly selected from the year group and snowball sampling was employed subsequently to recruit additional participants. A further twelve participants aged 14-18 years were recruited from youth groups, via leaders of the youth group, to ensure diversity in the sample in relation to socio-demographic characteristics and to enable data collection outside of the school environment. The sample included individuals who had, and hadn't, consumed alcohol. Parental consent and participant assent were given prior to participation. Participants aged over 16 years gave informed consent. Recruitment took place between January and October 2017. The number of participants recruited was determined by the point at which saturation was reached.

Data collection

A total of 36 semi-structured interviews were conducted (15 males, 21 females), three of which were paired interviews, and one of which was a triad interview, conducted with friends. Interviews were conducted in meeting rooms in schools (n=30) and youth groups (n=6). One focus group (n=6 males) was also conducted in a youth group. Interviews were conducted by GJM (PhD), a female researcher with training in qualitative research. No relationship was established with study participants before commencement of data collection. Before starting the interview, participants were aware of the

For peer review only - http://bmjopen.bmj.com/site/about/guidelines.xhtml

reasons for doing the research and the interviewer's aim of developing a preventive intervention in the future. Interviews were facilitated by a topic guide, which was used flexibly so that participants could explore views and opinions which were meaningful to them. The topic guide included questions around attitudes and perspectives around drinking, personal histories of alcohol use, risks and consequences of drinking, the role and influence of friendship groups and social networks, and family-related factors. Views on alcohol-related education and alcohol-related interventions were discussed but will be reported elsewhere. Interviews lasted between 30 and 60 minutes and were audio-recorded and transcribed verbatim. Interview data were anonymised. Participants received a £15 gift voucher for taking part.

Data analysis

Transcripts of interviews were imported into NVivo version 10 (QSR International, Brisbane) and analysed thematically using this software by GJM. Participants did not check or correct transcripts. Analysis aimed to capture emergent concepts and thus took an inductive approach. Transcripts were read and re-read and open line-by-line coding was conducted with a focus on understanding and capturing participants' experiences, behaviours, feelings, attitudes, and perceptions around alcohol use. We sought to explore relationships and patterns both within and between accounts. Different accounts were compared and contrasted, such that codes were progressively refined, and connections mapped, to characterise relationships and identify underlying concepts and then categories and themes. Ongoing engagement with the data ensured that emerging concepts were grounded in participants' accounts. Throughout the process the researcher wrote analytic memos and notes to capture thoughts around meanings, themes and relationships between themes. Thematic patterns were considered within and across cases and between groups of cases e.g. those who did, or did not, drink alcohol.

For peer review only

Sociological theories relating to social influences, group identity and social norms were explored during analysis to enhance understanding around the theoretical basis of influences on the initiation and maintenance of alcohol use behaviour evident from the data.(29-31) We also explored whether social practice theory (32-34) might help to explain alcohol use behaviour as a collective social practice during adolescence. However, while a social practice perspective helped to explain the social context of alcohol use and the meanings associated with alcohol consumption, it did not fully explain data regarding peer influences and/or pressures, which were key themes in our study.

Ethical considerations

Ethical approval for the study was obtained from the University of Bristol Faculty of Health Sciences Ethics Committee (study 131443 (8201)/2306).

Public involvement

We did not involve young people in this study directly, however, the authors engaged with young people advisory groups prior to commencing the programme of research, of which this is one part, and we have engaged with such advisory groups in relation to intervention development which builds on the findings reported in this paper.

For peer review only

Results

The major themes that emerged from the data were: normalisation and the wider culture, motivations, peer influence, pressure among peers, social media, and young people's drinking culture. Family influences were also a major theme, but we have not addressed them here, as we have previously reported similar findings elsewhere.(10)

Normalisation of drinking and the wider alcohol culture

Young people described alcohol consumption as a normalised practice and an accepted part of the cultural context. Young people accepted drinking as part of life and stressed the importance of autonomy around people's decisions regarding alcohol consumption. Exposure to teen films and social media presented alcohol consumption as fun and 'cool' and a means of enhancing popularity, while lacking details of negative consequences. Young people therefore frequently came into adolescence with clear pre-formed positive attitudes towards drinking and an understanding of its meanings in the social world (Supplementary material S1, quotes 1 and 2).

> *So it's just like you just grow up with like people around you like just drinking casually like with meals and stuff so no-one sees it as particularly dangerous … until like something actually goes wrong. Yeah. So you're kind of shielded from the the actual dangers… (ID 17, F)*

Reflecting the assimilation of wider cultural norms, perceptions of what constituted reasonable alcohol use involved levels of consumption far above levels at which drinking would become hazardous or harmful (see also S1, quote 3).

> *INT: And how much do you think is ok would you say?*
>
> *RES: Not getting drunk like really drunk like you can't even walk (ID 29, M)*

For peer review only - http://bmjopen.bmj.com/site/about/guidelines.xhtml

Despite such misperceptions, participants were aware of the balance between consuming alcohol for enjoyment and negative consequences, evidencing a clear awareness of the potential for a range of negative consequences to ensue if limits were overstepped (S1, quotes 4 and 5).

Motivations to drink and the initiation of alcohol use

Drinking became an integral practice in young people's social worlds by age 14-16. Young people most frequently described a shared meaning of alcohol consumption relating to collective fun, enjoyment and being cool. Drinking also improved confidence, popularity, and engagement with friends while enabling young people to rebel, make memories, and experiment, while providing a means of managing mental illness, avoiding reality, excusing behaviour or escaping stress (S2, quotes 1 and 2).

Satisfying the range of motivations to drink was straightforward since alcohol was both readily available and cheap. Some described initiation of drinking among peer groups via an influential individual, who was the first to provide or drink alcohol (often via parents), setting an expectation of drinking at subsequent parties.

> *RES: I think first party was in October when it was like, oh there's going to be alcohol there. .. And that was sort of an initiation. [LAUGHS] I don't know.*
>
> *INT: Yeah.*
>
> *RES: But it's sort of like the – yes it's after that party then it's sort of expected that there is at other ones, maybe. (ID 8, F)*

For peer review only - http://bmjopen.bmj.com/site/about/guidelines.xhtml

Young people's drinking and party culture

Drinking practice was rooted in house parties, which were viewed as a safer and more controlled setting than were outdoor settings. Parties' meanings were centred around the inherently social nature of the events, providing opportunities for socialising, shared experiences and a sense of togetherness with friends, as well as opportunity for drunkenness, which was a widely accepted norm. For many, alcohol made parties more enjoyable (S3, quote 1) and getting drunk provided an aim and purpose, while providing the means of looking popular, 'hard', or cool. Thus, widely held understandings, or aims, were to drink and 'get smashed' (S3, quotes 2 and 3), with the pace and extent of drunkenness often being a source of direct or indirect competition. Participants also described indirect pressures involving stigmatising insults linked to competence around intoxication.

> *RES: ..it's kind of like who can drink more and not get drunk, but it's like not a competition in that way, yeah.*
>
> *INT: So it's not a competition?*
>
> *RES: No, it's not a competition, but it feels like that because after you're a bit, oh, this guy's a lightweight, and that. (ID 37, M)*

Thus, it was important to tread a line between appearing competent in tolerating alcohol to avoid stigmatising labelling as a 'lightweight' (S3, quote 4), but to remain in control to avoid ruining a party and/or require care from friends, which was associated with reputational risk and disgruntlement from friends. Those who often took on the role of looking after friends described feelings of reluctance and resentment around missing out on enjoyment, or concern around managing the effects of intoxication.

> *The first feeling is panic and then you feel just disappointed and you're just angry. You feel… I feel a little bit angry but especially if they've done it before you know what you've done, you know how like what your limit is and yet you go out of your way to do it.. (ID 15, F)*

For peer review only - http://bmjopen.bmj.com/site/about/guidelines.xhtml

The disparity between individual autonomy in decisions around intoxication and the requirement for help from others thereafter was also viewed negatively (S3, quote 5).

Social influences on adolescent alcohol use

1. Peer influence

Social influences became paramount in mid-adolescence, with young people frequently reporting a desire to 'fit in' with friends. Those who drank earlier and to a greater extent acted as influential 'role models' for drinking (S4.1, quote 1). Thus, young people implicitly understood the social significance of joining in and facilitating belonging in the social world to enhance social status or to avoid social sanctions.

> *Kind of like 'cause everyone else is around you drinking, you kind of feel like you have to otherwise you don't really fit in um so that's what kind of happens to me most of the time. (ID 27, F)*

Social influences also facilitated a rise in the prevalence of drinkers, thus enhancing proximal social influences and further contributing to the normalisation of alcohol consumption. Social influence led to perceived requirements to join in in some way, and those who didn't join in with the vibe of the party were a 'buzz kill' (S4.1, quote 2). As such, a somewhat circular effect sustained the party culture, whereby the increasing prevalence of drinking strengthened group norms and social learning processes that generated a desire to drink to feel included, and which generated a social environment in which abstaining could be alien (see also S4.1, quote 3).

> *I didn't intend to drinking alcohol last year … but it just kind of happens, like when you're invited to a party you go just go and there's alcohol and you get, you get told there's going to be alcohol, and you just drink it to have a good time anyway 'cause everyone else is. (ID 39, M)*

For peer review only - http://bmjopen.bmj.com/site/about/guidelines.xhtml

Those who didn't comply with norms described feelings of awkwardness, embarrassment or boredom, or they avoided parties altogether, while others were incentivised to drink to avoid having to look after intoxicated friends (S4.1, quotes 4-6).

Notably, however, peer influences could have counter-effects, such that participants described alcohol consumption as pointless, expressing confusion around friends' drinking practices, while others reported aversion to role models of intoxication, which could strengthen views around abstinence (see also S4.1, quote 7).

> *I wouldn't do it because I have seen what in the middle, I have seen people feeling sick, all in the bath, like going on the street, I was like no way I am not doing that, you see what the impacts are if you drink. You see what would happen and I don't want that at all. (ID 6, F)*

Such individuals were less compliant with the group norm and often played a greater 'mum' role in supporting and helping others within the social group (S4.1, quote 8).

Individuals who abstained from drinking described the importance of friends with similar attitudes and behaviours around drinking and there was some evidence for peer selection in this group. Such friends provided support for their viewpoint and provided some protection from felt pressure or exclusion, enabling them to abstain and/or to enjoy social events more.

> *..if I was on my own it would have been worse, because you're just looking around and everyone's drinking. Not that it would lead me to drinking, but it's still better that I had friends who just don't do that at all: it made it much more fun (ID 16, M)*

For peer review only - http://bmjopen.bmj.com/site/about/guidelines.xhtml

Those who abstained frequently described strong interests in extra-curricular activities, particularly sports, a strong religious faith, and/or clear core values and aligned family viewpoints, which altered the meaning of alcohol use and reduced the effects of social influences and norms (S4.1, quote 9).

2. The influence of social media

The use of social media was integral to adolescent drinking behaviour and practice, particularly use of Instagram or the multimedia messaging app Snapchat. Posting pictures and videos from parties reflected the meanings associated with alcohol use, thus portraying participants being cool and sociable, to enhance popularity and social status. The ubiquity and prominence of social media weaved into young people's lives a relatively consistent, positive view of alcohol consumption that could act as another source of social influence and a visual depiction of norms that could contribute to a felt pressure to drink (see also S4.2, quotes 1 and 2).

> *Because in a friendship it's more like direct, like they're going to tell you that, but like, on a big scale like social media it kind of like tells you like that you should be drinking, like when you can, and the norm is to, and then I think you feel a bit pressured into it. (37, M)*

Social media used to demonstrate social capital, drinking 'competence' and maturity. Some were described as sipping drinks just to look right in pictures, while others aimed to depict their prowess in consumption.

> *I've noticed that a lot in my year group and like they'll kind of just… even if they don't really want to drink it they'll kind of just hold it there and they'll just like sip and stuff so they can have it for photos. (ID 34, F)*

Nevertheless, views on the impact on social media were mixed. A minority expressed caution about posting images of themselves drinking owing to potential reputational risk. A minority also reported

For peer review only - http://bmjopen.bmj.com/site/about/guidelines.xhtml

a view that drinking was unaffected by social media, although it was nevertheless described as a key component of drinking practice.

The pressurised environment

The combination of social media, social influences and social learning, alongside the meanings associated with drinking (e.g. belonging, popularity) and wider cultural influences, created a pressurised environment around drinking (Figure 1). Thus, the very interest in fitting in or feeling a need to join in with friends was felt to be subtly pressurising, while being the only person not drinking felt awkward and created unease, further contributing to a felt pressure.

> *Because you see everyone's doing it then you felt .. er like obliged to do it as well sometimes. Um I don't often, like, myself but you can definitely tell other people um it's like, oh everybody else is doing it so then I have to do it as well to fit in, it's like one of those sorts of things and also if your friends are drunk then it sort of becomes a thing where oh do you want mine you can have some, have some, and then you're like oh my gosh like do I accept, do I not (ID 15, F)*

Branding of individuals as 'wimps' or awarding 'lad points' for intoxication associated with courage and humour, further created incentives and obligations to drink to excess (S5, quote 1). The inextricable link between drinking alcohol and socialising meant that resisting pressures or challenging friends risked losing credibility as an integral and fun group member, or risked jeopardising friendships (see also S5, quotes 2 and 3).

> *INT: .. and how does that make you feel if you are saying no?*
>
> *RES: Um if you're like receiving yeah then you can kind of feel like it makes you not want to be in that situation, it makes you just like feel oh I don't feel safe in this environment anymore cos now I'm doing something that I don't want to do and you're just like what do I do, I can't deal with this situation and you don't want to*

For peer review only - http://bmjopen.bmj.com/site/about/guidelines.xhtml

For peer review only

not be friends with people who you're friends with, you don't want to break friendships over it but it's one of those things where you kind of if you don't like it then you have to say no and if your friends didn't accept that then it's like you have to sort that out with your friends yourself, that sort of thing. (ID 15, F)

Pressure to drink was felt in a range of ways, including subtle and indirect pressure through offers of alcoholic drinks, the felt need to be fun and to fit in, or proximal social influence. However, participants also reported more direct pressure through goading or direct insults, leading some to describe feeling 'forced to drink' and others wishing to avoid appearing cowardly. Overall, the social environment was felt to be conducive to feeling obliged to drink, or being 'pushed in the direction' towards alcohol use (see also S5 quotes 4 and 5).

.. I used to think alcohol was really bad but I guess as like it becomes more talked about, you're kind of dragged into it.

INT: Mm. It's interesting you say dragged into it.

RES: Not dragged – I'd say like kind of um pushed in the direction. (ID 27, F)

Nevertheless, such felt pressures were often unclear to the friends in question who were drinking, who therefore pressurised others unknowingly. Drinkers did not consider their actions to be pressurising, rather a gesture to enable or encourage friends to join in (see also S5, quote 6).

They just think they're saying it does – it's just a laugh like, if they're – they're kind of – they're almost trying to make their experience more enjoyable by saying if you do this… .. it will make it better, but I don't think that's the same kind of response for the person on the other end. (ID 33, M)

As outlined above, for non-drinkers, having friends with a similar viewpoint provided support and reduced felt pressure (S5, quote 7).

For peer review only - http://bmjopen.bmj.com/site/about/guidelines.xhtml

Discussion

In this paper, we have demonstrated how the wider alcohol culture normalised alcohol use for young people, helping to generate perceptions of drinking as a practice associated with being cool, mature and popular as well as an important means of enhancing enjoyment and social status. The ubiquity of alcohol use generated normative social influences and opportunities for social learning around initiation and continuation of alcohol use. Together with embedded meanings and expectancies, the interaction between peer norms, social influences and the effects of social media generated a pressurised environment in which young people felt obliged to comply and join in.

The strengths of our study include the gender-balanced sample of young people from both schools and youth groups, including those who used alcohol and those who abstained. We also used one-to-one interviews, which provided space for young people to share their views around drinking, without concerns around disagreement or judgement from peers or friends. A small proportion of interviews were conducted with dyads with one triad, however, we did not find evidence that participants exaggerated or withheld views and perspectives in these pairs, and paired interviews were conducted when expressed as a preference by the participants. Nevertheless, our data were not triangulated, and participants were recruited from urban and suburban settings only, so our findings cannot necessarily be generalised to different geographical or demographic contexts. Interestingly, although parental messages and the off-putting effect of observing intoxicated role models were described by some moderate drinkers, our data did not provide evidence regarding factors that might be affecting downward trends in the prevalence of alcohol use among young people in England, and further qualitative and quantitative research will be needed to explore such trends. Lastly, while we have reported that social influences and pressures are a critical influence on young people's alcohol use, further research will be needed to explore exactly how such influences could be effectively addressed in public health interventions for young people.

For peer review only - http://bmjopen.bmj.com/site/about/guidelines.xhtml

The frequently reported desire to fit in with the group, and unease expressed in relation to abstinence, highlighted the central role of normative social influence in generating a collective practice in which intoxication was accepted and expected. Evidence suggests that both direct and indirect pathways of peer influences and pressures may exist, which can contribute to generating social influences (and can act together with peer selection (19, 35)). Pathways can be direct, including offers of drinks, dares, or goading, and indirect, including modelling of peer drinking and/or contributions to the formation of norms, beliefs and expectancies,(36, 37) which were also evident in our study. In addition, studies have reported an impact of being around peers who drink alcohol, either through proximity to social acquaintances who consume alcohol or through time spent with drinking peers,(38, 39) highlighting the important impact of the rise in prevalence of alcohol use through adolescence. This was evident in our study since being around other drinkers and being immersed in a practice in which drinking was highly prevalent (contributing to descriptive norms) contributed to the felt obligation to drink.

Frequent reports by young people of needing to 'fit in' suggest that injunctive norms, or perceptions of peer approval of drinking, were also critical. Thus, our findings support those of quantitative studies, which have reported associations between both descriptive and injunctive norms and frequency and quantity of adolescence alcohol use and heavy episodic drinking,(40, 41). Specifically, one study reported a greater impact of injunctive compared to descriptive norms, with the former associated with a range of measures of alcohol use as well as alcohol-related negative consequences.(41)

In addition to social norms and influences among peers, social media, or social networking sites (SNS), played a role in providing a physical depiction of positive alcohol-related descriptive and injunctive norms, which was an integral part of the social practice of adolescent alcohol use. Thus, we have reported evidence to support a previous study describing 'intoxigenic digital identities' and

For peer review only - http://bmjopen.bmj.com/site/about/guidelines.xhtml

‘intoxigenic digital spaces’ that contribute to the normalisation of alcohol use.(20) Similarly, a qualitative study of young women’s drinking cultures described the critical nature of SNS in facilitating displays of popularity, belonging, membership of the social network and ‘friendship fun’.(22) Such media also bring about opportunities for misperceptions owing to the circulation of positive imagery which can be interpreted at face value or as ‘truth’.(23, 42) Quantitative data have demonstrated an association between exposure to friends’ alcohol-related SNS content and initiation of alcohol use and stronger pro-alcohol peer injunctive norms, highlighting how the provision of opportunities for observation, comparison and role-modelling of behaviours through SNS can impact on norm perception and behaviour.(24) In addition, others have reported an association between alcohol promotion through digital media and adolescent alcohol consumption.(43, 44)

Taken together, therefore, evidence suggests the need for public health interventions to effectively address both peer norms and social influences during adolescence, including the effects of SNS, to enhance the likelihood of effective prevention. Furthermore, the evidence of felt pressure resulting from the influences discussed above suggests that many young people feel a lack of autonomy around alcohol consumption, and constraint around behavioural decisions within their social groups. Thus, in addition to addressing norm perception and normative social influence, there is a need for interventions to find ways of overcoming such constraints and fostering greater resistance to conformity. In part, this may require strengthening the voice and influence of those in the minority group (i.e. those who abstain from drinking) as outlined in Moscovici’s theory of minority influence (45, 46), which may be increasingly feasible in light of reported downward trends in adolescent alcohol consumption (3). It has also been suggested that feelings of powerlessness during adolescence may be overcome through enhanced social status for young people, for instance through opportunities for creative activities and active engagement in communities.(47)

For peer review only - http://bmjopen.bmj.com/site/about/guidelines.xhtml

Conclusions

Our study has demonstrated the effect of the interplay between cultural norms, social influences, social pressures, and social media, particularly the critical role of normative social influence, in driving the escalation and normalisation of alcohol use in mid-adolescence. Our findings suggest that there is a need for a combination of approaches to effectively prevent excessive alcohol use among young people. First, population- and family-level interventions are needed to address pro-alcohol cultural norms. Second, interventions in young people need to target normative social influence and peer norms to interrupt the rise in prevalence of excessive drinking, while aiming to enhance social status and the ability to challenge social pressures, to increase the likelihood of preventing alcohol-related harm.

For peer review only

Acknowledgements

GJM was supported by an NIHR post-doctoral fellowship award (PDF-2013-06-026). This report is independent research supported by the National Institute for Health Research (Post-Doctoral Fellowship, PDF-2013-06-026). The views expressed in this publication are those of the author(s) and not necessarily those of the NHS, the National Institute for Health Research or the Department of Health.

The work was undertaken with the support of The Centre for the Development and Evaluation of Complex Interventions for Public Health Improvement (DECIPHer), a UKCRC Public Health Research Centre of Excellence. Joint funding (MR/KO232331/1) from the British Heart Foundation, Cancer Research UK, Economic and Social Research Council, Medical Research Council, the Welsh Government and the Wellcome Trust, under the auspices of the UK Clinical Research Collaboration, is gratefully acknowledged.

RC is supported by the University of Bristol.

We are extremely grateful to the schools, youth groups and participants for their assistance and participation in this study. We would also like to thank Bristol Drugs Project for their assistance with our work.

For peer review only

For peer review only - http://bmjopen.bmj.com/site/about/guidelines.xhtml

References

1. Looze Md, Raaijmakers Q, Bogt Tt, Bendtsen P, Farhat T, Ferreira M, et al. Decreases in adolescent weekly alcohol use in Europe and North America: evidence from 28 countries from 2002 to 2010. European Journal of Public Health. 2015;25(suppl_2):69-72.
2. Kraus L, Seitz, N-N, Piontek D, Molinaro S et al,. "Are The Times A-Changin'"? Trends in Adolescent Substance Use in Europe. Addiction. 2018.
3. Pape H, Rossow I, Brunborg Geir S. Adolescents drink less: How, who and why? A review of the recent research literature. Drug and Alcohol Review. 2018;37(S1):S98-S114.
4. NHS Digital. Smoking, drinking and drug use among young people. England 2016. UK.: National Statistics; 2017.
5. Public Health England. Data intelligence summary: Alcohol consumption and harm among under 18 year olds. UK.: Public Health England.; 2016. Contract No.: Publications gateway number: 2016173.
6. Department of Health. Guidance on the consumption of alcohol by children and young people. Report from Sir Liam Donaldson, Chief Medical Officer for England. London: Department of Health; 2009.
7. Rossow I, Keating P, Felix L, McCambridge J. Does parental drinking influence children's drinking? A systematic review of prospective cohort studies. Addiction. 2016;111(2):204-17.
8. Mattick RP, Clare, Philip J., Aiken, Alexandra, Wadolowski, Monika, Hutchinson, Delyse, Najman, Jackob, Slade, Tim, Bruno, Raimondo, McBride, Nyanda, Kypri, Kypros, Vogl, Laura, Degenhardt, Louisa. Association of parental supply of alcohol with adolescent drinking, alcohol-related harms, and alcohol use disorder symptoms: a prospective cohort study. The Lancet Public Health. 2018;3(2):e64-e71.
9. Özdemir M, Koutakis N. Does promoting parents' negative attitudes to underage drinking reduce adolescents' drinking? The mediating process and moderators of the effects of the Örebro Prevention Programme. Addiction. 2016;111(2):263-71.
10. Jacob N, MacArthur GJ, Hickman M, Campbell R. A qualitative investigation of the role of the family in structuring young people's alcohol use. The European Journal of Public Health. 2015.
11. Campbell JM, Oei TP. A cognitive model for the intergenerational transference of alcohol use behavior. Addictive Behaviors. 2010;35(2):73-83.
12. Mahedy L, MacArthur, GJ, Hammerton, G, Edwards, AC, Kendler, KS, Macleod, J, Hickman, M, Moore, SC, Heron J. The effect of parental drinking on alcohol use in young adults: the mediating role of parental monitoring and peer deviance. Addiction.epub.:doi: 10.1111/add.14280.
13. MacArthur GJ, Jacob N, Pound P, Hickman M, Campbell R. Among friends: a qualitative exploration of the role of peers in young people's alcohol use using Bourdieu's concepts of habitus, field and capital. Sociology of Health & Illness. 2016:n/a-n/a.
14. de Visser RO, Wheeler Z, Abraham C, Smith JA. 'Drinking is our modern way of bonding': Young people's beliefs about interventions to encourage moderate drinking. Psychology & Health. 2013;28(12):1460-80.
15. Niland P, Lyons AC, Goodwin I, Hutton F. "Everyone can loosen up and get a bit of a buzz on": Young adults, alcohol and friendship practices. International Journal of Drug Policy. 2013;24(6):530-7.
16. Kelly AB, Chan GCK, Toumbourou JW, O'Flaherty M, Homel R, Patton GC, et al. Very young adolescents and alcohol: evidence of a unique susceptibility to peer alcohol use. Addictive Behaviors. 2012;37(4):414-9.
17. Ali MM, Dwyer DS. Social network effects in alcohol consumption among adolescents. Addictive behaviors. 2010;35:337-42.

For peer review only - http://bmjopen.bmj.com/site/about/guidelines.xhtml

18. Percy A, Wilson, J., McCartan,C., McCrystal, P. Teenage drinking cultures. York: Joseph Rowntree Foundation; 2011.
19. Leung RK, Toumbourou JW, Hemphill SA. The effect of peer influence and selection processes on adolescent alcohol use: a systematic review of longitudinal studies. Health Psychology Review. 2014;8(4):426-57.
20. Griffiths R, Casswell S. Intoxigenic digital spaces? Youth, social networking sites and alcohol marketing. Drug and Alcohol Review. 2010;29(5):525-30.
21. Goodwin I, Griffin C, Lyons A, McCreanor T, Barnes HM. Precarious Popularity: Facebook Drinking Photos, the Attention Economy, and the Regime of the Branded Self. Social Media + Society. 2016;2(1):2056305116628889.
22. Atkinson AM, Sumnall HR. 'If I don't look good, it just doesn't go up': A qualitative study of young women's drinking cultures and practices on Social Network Sites. International Journal of Drug Policy. 2016;38:50-62.
23. McCreanor T, Lyons A, Griffin C, Goodwin I, Moewaka Barnes H, Hutton F. Youth drinking cultures, social networking and alcohol marketing: implications for public health. Critical Public Health. 2012;23(1):110-20.
24. Nesi J, Rothenberg WA, Hussong AM, Jackson KM. Friends' Alcohol-Related Social Networking Site Activity Predicts Escalations in Adolescent Drinking: Mediation by Peer Norms. Journal of Adolescent Health.
25. Townshend TG. Youth, alcohol and place-based leisure behaviours: A study of two locations in England. Social Science & Medicine. 2013;91(0):153-61.
26. Bremner P, Burnett, J., Nunney, F., Ravat, M., Mistral, W. Young people, alcohol and influences. A study of young people and their relationship with alcohol. York; 2011.
27. Scott S, Shucksmith J, Baker R, Kaner E. 'Hidden Habitus': A Qualitative Study of Socio-Ecological Influences on Drinking Practices and Social Identity in Mid-Adolescence. International Journal of Environmental Research and Public Health. 2017;14(6):611.
28. Larm P, Livingston, M., Svensson, J., Leifman, H., Raninen, J. The increased trend of non-drinking in adolescence: The role of parental monitoring and attitudes toward offspring drinking. Drug and Alcohol Review. 2018;37(S1):S34-S41.
29. Turner JC. Social Influence. Buckingham: Open University Press; 1991.
30. Tajfel H, Flament, C., Billig, M.G. et al,. Social categorisation and intergroup behaviour. European Journal of Social Psychology. 1971;5:245-53.
31. Sherif M. The psychology of social norms. New York.: Harper and Brothers; 1936.
32. Blue S, Shove E, Carmona C, Kelly MP. Theories of practice and public health: understanding (un)healthy practices. Critical Public Health. 2016;26(1):36-50.
33. Shove E. PM, Watson M.,. The Dynamics of Social Practice: Everyday Life and How it Changes. London: Sage; 2012.
34. Meier PS, Warde A, Holmes J. All drinking is not equal: how a social practice theory lens could enhance public health research on alcohol and other health behaviours. Addiction. 2018;113(2):206-13.
35. Osgood DW, Ragan DT, Wallace L, Gest SD, Feinberg ME, Moody J. Peers and the Emergence of Alcohol Use: Influence and Selection Processes in Adolescent Friendship Networks. Journal of Research on Adolescence. 2013;23(3):500-12.
36. Borsari B, Carey KB. Peer influences on college drinking: A review of the research. Journal of Substance Abuse. 2001;13(4):391-424.
37. Studer J, Baggio S, Deline S, N'Goran AA, Henchoz Y, Mohler-Kuo M, et al. Peer pressure and alcohol use in young men: A mediation analysis of drinking motives. International Journal of Drug Policy. 2014;25(4):700-8.
38. Dallas R, Field M, Jones A, Christiansen P, Rose A, Robinson E. Influenced but Unaware: Social Influence on Alcohol Drinking Among Social Acquaintances. Alcoholism: Clinical and Experimental Research. 2014;38(5):1448-53.

For peer review only - http://bmjopen.bmj.com/site/about/guidelines.xhtml

39. Salvy S-J, Pedersen ER, Miles JNV, Tucker JS, D'Amico EJ. Proximal and distal social influence on alcohol consumption and marijuana use among middle school adolescents. Drug and Alcohol Dependence. 2014;144:93-101.
40. Song E-Y, Smiler AP, Wagoner KG, Wolfson M. Everyone Says It's OK: Adolescents' Perceptions of Peer, Parent, and Community Alcohol Norms, Alcohol Consumption, and Alcohol-Related Consequences. Substance Use & Misuse. 2012;47(1):86-98.
41. Pedersen ER, Osilla KC, Miles JNV, Tucker JS, Ewing BA, Shih RA, et al. The role of perceived injunctive alcohol norms in adolescent drinking behavior. Addictive Behaviors. 2017;67:1-7.
42. Moreno MA, Briner LR, Williams A, Brockman L, Walker L, Christakis DA. A Content Analysis of Displayed Alcohol References on a Social Networking Web Site. Journal of Adolescent Health.47(2):168-75.
43. Lobstein T, Landon J, Thornton N, Jernigan D. The commercial use of digital media to market alcohol products: a narrative review. Addiction. 2017;112:21-7.
44. Scott S, Muirhead C, Shucksmith J, Tyrrell R, Kaner E. Does Industry-Driven Alcohol Marketing Influence Adolescent Drinking Behaviour? A Systematic Review. Alcohol and Alcoholism. 2017;52(1):84-94.
45. Moscovici S, Personnaz, B.,. Studies in Social Influence. V. Minority Influence and Conversion Behavior in a Perceptual Tast. Journal of Experimental Social Psychology. 1980;16:270-82.
46. Moscovici S, Lage E. Studies in social influence III: Majority versus minority influence in a group. European Journal of Social Psychology. 1976;6(2):149-74.
47. Pound P, Campbell R. Locating and applying sociological theories of risk-taking to develop public health interventions for adolescents. Health Sociology Review. 2015;24(1):64-80.

For peer review only

For peer review only - http://bmjopen.bmj.com/site/about/guidelines.xhtml

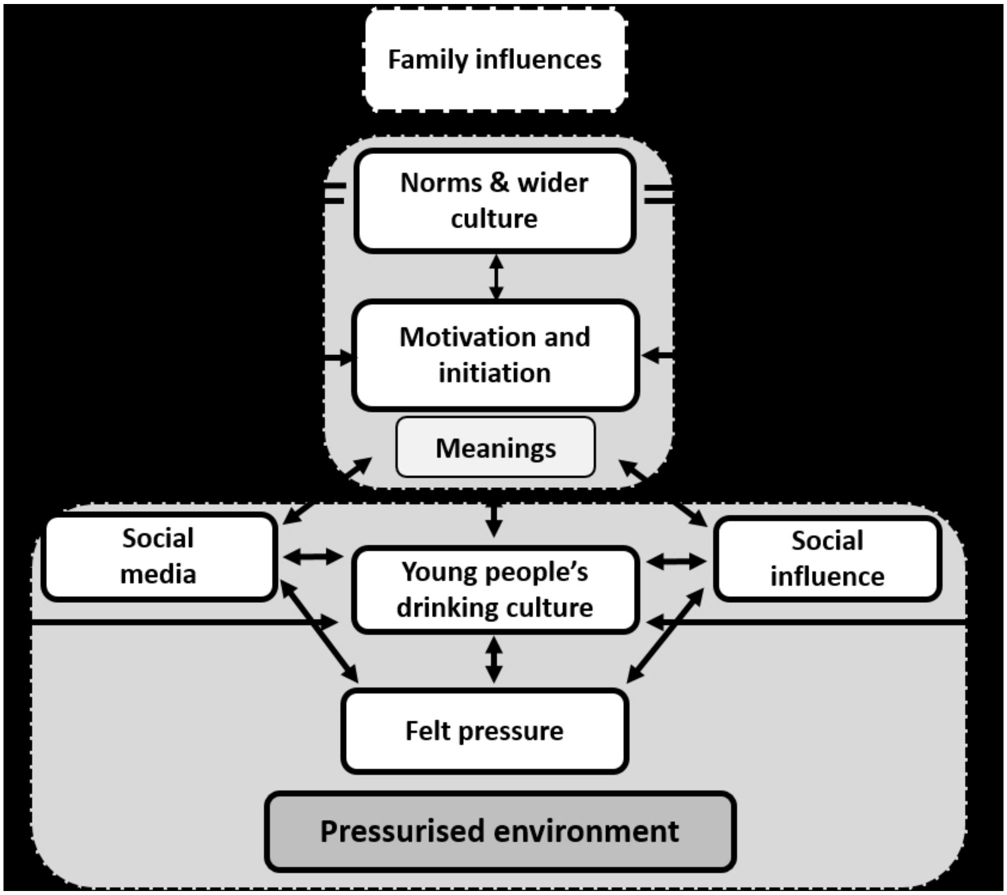

Intersection of social and cultural influences on adolescent alcohol use and generation of a pressurised environment. Note that family influences have been addressed elsewhere.

Supplementary Material: Additional quotes

S1. Normalisation of drinking and the wider alcohol culture

1. *Yes but also like in films and like series, and social media, parties just look like crazy cus everyone's like getting drunk, so people who kind of their aim is, I wanna be cool, I wanna have lots of friends, and I wanna be the popular one. They think, the only way to do that is to go to the parties, cus that's what all the films say. (ID 13b, F)*

2. *I didn't really know that what it can actually do to you. I just thought oh it makes you happy and that's it, that's all you really hear about. (ID 21, F)*

3. *RES: … I think drinking [sic: in adults] is fine because like obviously people really enjoy like wine and stuff, um, but I think it's bad when you're like addicted or like drinking more than is necessary. Yeah.*

   INT: And how much would that be do you reckon, the drinking more than you need to?

   RES: Um, like, I dunno, if someone was drinking like a bottle of wine a day that's like… that's just like someone who's like… it's like kind of like a normal amount to drink but it's a lot. But if someone's actually drunk throughout the day that's terrible I think. Yeah. (ID 17, F)

4. *Well there's a point where it's like, not fun anymore and also, it's just not cool anymore. It can be seen as like, cool having a few cans, getting drunk but then like after that it's just silly. And people will like, ignore you, in that situation (ID 13b, F)*

For peer review only - http://bmjopen.bmj.com/site/about/guidelines.xhtml

For peer review only

5. *INT:* *Yeah, and what might be some of the bad things that you mention there?*

RES: *Getting addicted or feeling sick the next day. You can do bad things when you're drunk.*

INT: *Bad things?*

RES: *Well you don't really know what you're doing. (ID 40, M)*

For peer review only

S2. Motivations to drink and the initiation of alcohol use

1. *.. you could forget your troubles maybe and just live in the moment and just go, 'I'll deal with the problems later. I'll have fun now'. (ID 22, M)*

2. *I think it's a good social thing like it makes it easier for you to talk to people, you know, and you can - you don't have to worry about like making a fool of yourself because you have an excuse and stuff. (ID 18, F)*

For peer review only

For peer review only - http://bmjopen.bmj.com/site/about/guidelines.xhtml

S3: Young people's drinking and party culture

1. *People are saying oh its so, it will be so dead like without them drinking, like it will just be so boring and stuff, but they say like oh its better when we are drinking because it's more fun and like everybody is falling and everybody is like laughing. (ID 6, F)*

2. *Yeah, some people like – some people might usually go into like say the party and stuff and just say like "Oh I wanna get smashed" or something like that. (ID 36, M)*

3. *It's just mainly like parties, I guess, like people just think, it's a party, you have to drink, basically. (35b, F)*

4. *I'm not like – people often call you, um, a lightweight when you drink and get drunk, and it's like that's an offensive term. It's quite embarrassing if you get called that, but yeah, I think there's a lot of stigma towards that. (ID 32, F)*

5. *RES: I could be their friend and then they could do something stupid and I'd have to look after them or go and try and fix it, um, yeah.*

   INT: And how would that make you feel doing that?

   RES: Quite annoyed because they got themselves like this. (ID 22, M)

For peer review only - http://bmjopen.bmj.com/site/about/guidelines.xhtml

For peer review only

S4. Social influences on adolescent alcohol use

4.1. Peer influence

1. *INT: And what do you think might have changed those people's opinion, how you said some people didn't and now do?*

   RES: Probably because they've seen the first people do it and once somebody…

   INT: Hmm.

   RES: … one person starts doing it and then the next person then I think eventually it becomes like, everyone just wants to try it and then if they like it then they'll do it again and again. (ID 34, F)

2. *Like one party we were at there's a group of people and they were talking about revision and so you just like walk away from them, and you're like 'oh, they're such like a buzz-kill' because it's not… they don't have to be drunk but they're just like changing… they're like shifting the… like the, um, like the vibe of the party to more like, I dunno, not as funny. (ID 17, F)*

3. *INT: What do you think are the main reasons people might drink?*

   RES: Um, just they're eager to drink, and their friends are drinking. They will just drink. Like, it's... I mean, sometimes it's not even peer pressure; it's just seeing other people doing it, and you just want to do it as well, just to fit in. (ID 16, M)

4. *I know definitely that I felt a bit like not left out but like, not part of it, that's why I didn't enjoy it. It was really boring cus like I didn't wanna talk to anyone because they were all just like really drunk. So I just stayed like half an hour. (ID 13 a, F)*

For peer review only - http://bmjopen.bmj.com/site/about/guidelines.xhtml

5. *RES: Maybe like if you're invited to a party and everyone else there. You don't really wanna be the one that...the only one not.*

INT: Um. How would it feel if you were the only person who wasn't do you think?

RES: Embarrassed maybe.

INT: Um and why do you think that is?

RES: Um 'cause you don't feel like you fit in the group. (ID 40, M)

6. *But I think it's more like yeah you don't want to be sober at a party if you don't want to be the person to look after all your friends.* (ID 21, F)

7. *Because they see people when they're drunk and they'll think well that… that may… may… you may think is fun but it really doesn't look that fun cos you're jumping about and jumping on your head and stuff like that. What's the point? (ID 22, M)*

8. *RES 1: .. in my friendship group I'm kind of like the mum like I'm always like.*

RES 2: Me too.

RES 1: I'm always like just making sure everyone's ok.

RES 2: Checking everyone's all right. (ID 31a F; 31b, F)

9. *I have had the invites from my friends but I've chosen not to go because I find that sport is more important to me, than just going drinking. (ID 37, M)*

4.2. The influence of social media

1. *They kind of make you feel like you want to drink more cus it's like they're having... When you look on Snapchat, most of the time, it just seems like you can see, like, the good things. Cos people filming it are like oh such good fun. They won't put on*

For peer review only - http://bmjopen.bmj.com/site/about/guidelines.xhtml

stuff that's like really bad. So it makes it; like I guess it gives the alcohol like a more positive view (ID 8b, F)

2. *RES: I think whenever people are having like a night, like party then there's always like drinks on their Snapchat which just shows that they're having fun and they're drinking…*

INT: Mm.

RES: So I wanna do that. And go to more things like that. (ID 26, F)

For peer review only

S5. The pressurised environment

1. *INT: What kind of things would people say? What sort of um, type of thing would they be?*

   RES: Just like – like calling them scared and stuff for not doing it and stuff like that.

   INT: Okay. Yeah. Yeah.

   RES: Like, are you too scared to do this? But like other words. (ID 33, M)

2. *.. a lot of people drink to impress their friends so say like one friends like oh yeah I can drink this much and then the others like well I can drink this much and it's almost like a sly competition and then that's when it gets bad and out of hand like I've seen that from friends and then they've lost friends like because of it. (ID 31a, F)*

3. *RES: I think it's harder because what people want other people's – they want other people to think about them like in a more positive way like…*

   INT: Hmm.

   RES: … they don't wanna be thought of this person who's like not fun and doesn't want to do all the stuff… (ID 33, M)

4. *RES3: Some people think you have to, just like peer pressure, I guess, if you're at a party or something [laughs].*

   RES2: Mm.

   INT: What does that kind of feel like? What happens, when you say, 'peer pressure', what's...?

   RES1: Forced into doing it, really. (ID 35a (M), b (F), c (F))

For peer review only - http://bmjopen.bmj.com/site/about/guidelines.xhtml

5. *RES: I think they always teach like I think they always teach peer pressure wrong.*

INT: Right. So what do they say?

RES: I mean they - well they always just say like if someone asks you to do something that you don't want to do just say no. But it-it's not really that. I think it's more that people are doing things and if you are not part of it – well you're not – like if you're not doing it as well you're not part of it... (ID 7, F)

6. *RES: I don't think so. I think I haven't really ever experienced any, like, peer pressure. Um, but I still think there is quite a lot of pressure to drink, um, whether or not anyone means to pressurise you. (ID 32, F)*

7. *.. I don't know if I didn't have my friends or anyone to tell me not to drink, I'd probably be drinking by now. .. So, I'm kind of happy that they're around. (ID 12, M)*

Abbreviations:

INT: Interviewer

RES: respondent

For peer review only

COREQ (COnsolidated criteria for REporting Qualitative research) Checklist

A checklist of items that should be included in reports of qualitative research. You must report the page number in your manuscript where you consider each of the items listed in this checklist. If you have not included this information, either revise your manuscript accordingly before submitting or note N/A.

Topic	Item No.	Guide Questions/Description	Reported on Page No.
**Domain 1: Research team and reflexivity**			
Personal characteristics			
Interviewer/facilitator	1	Which author/s conducted the interview or focus group?	
Credentials	2	What were the researcher's credentials? E.g. PhD, MD	
Occupation	3	What was their occupation at the time of the study?	
Gender	4	Was the researcher male or female?	
Experience and training	5	What experience or training did the researcher have?	
Relationship with participants			
Relationship established	6	Was a relationship established prior to study commencement?	
Participant knowledge of the interviewer	7	What did the participants know about the researcher? e.g. personal goals, reasons for doing the research	
Interviewer characteristics	8	What characteristics were reported about the inter viewer/facilitator? e.g. Bias, assumptions, reasons and interests in the research topic	
**Domain 2: Study design**			
Theoretical framework			
Methodological orientation and Theory	9	What methodological orientation was stated to underpin the study? e.g. grounded theory, discourse analysis, ethnography, phenomenology, content analysis	
Participant selection			
Sampling	10	How were participants selected? e.g. purposive, convenience, consecutive, snowball	
Method of approach	11	How were participants approached? e.g. face-to-face, telephone, mail, email	
Sample size	12	How many participants were in the study?	
Non-participation	13	How many people refused to participate or dropped out? Reasons?	
Setting			
Setting of data collection	14	Where was the data collected? e.g. home, clinic, workplace	
Presence of non-participants	15	Was anyone else present besides the participants and researchers?	
Description of sample	16	What are the important characteristics of the sample? e.g. demographic data, date	
Data collection			
Interview guide	17	Were questions, prompts, guides provided by the authors? Was it pilot tested?	
Repeat interviews	18	Were repeat inter views carried out? If yes, how many?	
Audio/visual recording	19	Did the research use audio or visual recording to collect the data?	
Field notes	20	Were field notes made during and/or after the inter view or focus group?	
Duration	21	What was the duration of the inter views or focus group?	
Data saturation	22	Was data saturation discussed?	
Transcripts returned	23	Were transcripts returned to participants for comment and/or	

For peer review only

Topic	Item No.	Guide Questions/Description	Reported on Page No.
		correction?	
**Domain 3: analysis and findings**			
Data analysis			
Number of data coders	24	How many data coders coded the data?	
Description of the coding tree	25	Did authors provide a description of the coding tree?	
Derivation of themes	26	Were themes identified in advance or derived from the data?	
Software	27	What software, if applicable, was used to manage the data?	
Participant checking	28	Did participants provide feedback on the findings?	
Reporting			
Quotations presented	29	Were participant quotations presented to illustrate the themes/findings? Was each quotation identified? e.g. participant number	
Data and findings consistent	30	Was there consistency between the data presented and the findings?	
Clarity of major themes	31	Were major themes clearly presented in the findings?	
Clarity of minor themes	32	Is there a description of diverse cases or discussion of minor themes?	

Developed from: Tong A, Sainsbury P, Craig J. Consolidated criteria for reporting qualitative research (COREQ): a 32-item checklist for interviews and focus groups. *International Journal for Quality in Health Care*. 2007. Volume 19, Number 6: pp. 349 – 357

Once you have completed this checklist, please save a copy and upload it as part of your submission. DO NOT include this checklist as part of the main manuscript document. It must be uploaded as a separate file.

For peer review only

BMJ Open

A qualitative exploration of the intersection between social influences and cultural norms in relation to the development of alcohol use behaviour during adolescence.

Journal:	*BMJ Open*
Manuscript ID	bmjopen-2019-030556.R1
Article Type:	Original research
Date Submitted by the Author:	15-Jan-2020
Complete List of Authors:	MacArthur, George; Bristol University, Social and Community Medicine<br>Hickman, Matthew; Bristol<br>Campbell, Rona; University of Bristol, School of Social and Community Medicine
**Primary Subject Heading**:	Qualitative research
Secondary Subject Heading:	Public health
Keywords:	QUALITATIVE RESEARCH, PREVENTIVE MEDICINE, PUBLIC HEALTH

SCHOLARONE™
Manuscripts

I, the Submitting Author has the right to grant and does grant on behalf of all authors of the Work (as defined in the below author licence), an exclusive licence and/or a non-exclusive licence for contributions from authors who are: i) UK Crown employees; ii) where BMJ has agreed a CC-BY licence shall apply, and/or iii) in accordance with the terms applicable for US Federal Government officers or employees acting as part of their official duties; on a worldwide, perpetual, irrevocable, royalty-free basis to BMJ Publishing Group Ltd ("BMJ") its licensees and where the relevant Journal is co-owned by BMJ to the co-owners of the Journal, to publish the Work in this journal and any other BMJ products and to exploit all rights, as set out in our licence.

The Submitting Author accepts and understands that any supply made under these terms is made by BMJ to the Submitting Author unless you are acting as an employee on behalf of your employer or a postgraduate student of an affiliated institution which is paying any applicable article publishing charge ("APC") for Open Access articles. Where the Submitting Author wishes to make the Work available on an Open Access basis (and intends to pay the relevant APC), the terms of reuse of such Open Access shall be governed by a Creative Commons licence – details of these licences and which Creative Commons licence will apply to this Work are set out in our licence referred to above.

Other than as permitted in any relevant BMJ Author's Self Archiving Policies, I confirm this Work has not been accepted for publication elsewhere, is not being considered for publication elsewhere and does not duplicate material already published. I confirm all authors consent to publication of this Work and authorise the granting of this licence.

For peer review only

A qualitative exploration of the intersection between social influences and cultural norms in relation to the development of alcohol use behaviour during adolescence.

MacArthur GJ[1], Hickman M[1], Campbell R[1]

Running title: Adolescent alcohol use: norms and influences.

[1] Population Health Sciences, Bristol Medical School, University of Bristol, Canynge Hall, 39 Whatley Road, Bristol BS8 2PS, UK.

MacArthur GJ PhD, NIHR postdoctoral research fellow, Department of Population Health Sciences, University of Bristol

Hickman M PhD, Professor of Epidemiology and Public Health, Department of Population Health Sciences, University of Bristol

Campbell R PhD, Professor of Public Health, Department of Population Health Sciences, University of Bristol

Corresponding author:

Address: Population Health Sciences, Bristol Medical School, University of Bristol, Canynge Hall, 39 Whatley Road, Bristol BS8 2PS, UK.

Email: georgie.macarthur@bristol.ac.uk

Tel: +44 (0) 117 928 7295

For peer review only

For peer review only - http://bmjopen.bmj.com/site/about/guidelines.xhtml

Abstract

Objectives

Few contemporary studies have examined peer and social drivers of alcohol use during mid-adolescence. We sought to explore young people's perspectives on socio-cultural influences relating to alcohol use behaviour during this period.

Design

Qualitative research study

Methods

Semi-structured one-to one (n=25), paired (n=3) or triad (n=1) interviews and one focus group (n=6) were conducted with thirty young people aged 14-15 (13 males, 17 females) recruited from four schools, and twelve participants (aged 14-18, 8 males, 4 females) recruited from two youth groups in an urban centre in the West of England. Nineteen participants abstained from alcohol use, nine were occasional or moderate drinkers and fourteen drank alcohol more regularly. Interviews were audio-recorded, transcribed verbatim and analysed thematically using NVivo 10, through a lens of social influence and social norms theories.

Results

Alcohol consumption was associated with being cool, mature and popular, while enabling escape from reality and boosting confidence and enjoyment. Positive expectancies, alongside opportunity, contributed to motivating initiation, but social influences were paramount, with participants describing a need to 'fit in' with friends to avoid social exclusion. Such influences positioned drinking at parties as a normative social practice, providing opportunities for social learning and the strengthening of peer norms. Social media presented young people with positive alcohol-associated depictions of social status, enjoyment and maturity. This intersection of influences and norms generated a pressurised environment and a sense of unease around resisting pressures, which could elicit stigmatising insults.

Discussion and Conclusions

For peer review only - http://bmjopen.bmj.com/site/about/guidelines.xhtml

Cultural norms, social influences and social media intersect to create a pressurised environment around alcohol use during mid-adolescence, driving the escalation in the prevalence of excessive consumption at this stage. New interventions need to address normative influences to enable the prevention of excessive alcohol use during adolescence.

Strengths and Limitations of this study

1. Strengths of the study include the gender-balanced sample of young people from both schools and youth groups, including those who used alcohol and those who abstained.
2. One-to-one interviews were conducted, which provided space for young people to share their views around drinking, without concerns around disagreement or judgement from peers or friends.
3. Limitations of this study include the lack of triangulation of data, and participants were recruited from urban and suburban settings only, meaning that our findings cannot necessarily be generalised to different geographical or demographic contexts.

Funding statement

GJM was supported by an NIHR post-doctoral fellowship award (PDF-2013-06-026). This report is independent research supported by the National Institute for Health Research (Post-Doctoral Fellowship, PDF-2013-06-026). The views expressed in this publication are those of the author(s) and not necessarily those of the NHS, the National Institute for Health Research or the Department of Health and Social Care.

The work was undertaken with the support of The Centre for the Development and Evaluation of Complex Interventions for Public Health Improvement (DECIPHer), a UKCRC Public Health Research Centre of Excellence. Joint funding (MR/KO232331/1) from the British Heart Foundation, Cancer Research UK, Economic and Social Research Council, Medical Research Council, the Welsh

For peer review only - http://bmjopen.bmj.com/site/about/guidelines.xhtml

For peer review only

Government and the Wellcome Trust, under the auspices of the UK Clinical Research Collaboration, is gratefully acknowledged.

RC is supported by the University of Bristol.

Competing interests

GJM declares no competing interests.

RC is a scientific advisor to Evidence to Impact a not-for-profit company wholly owned by the Universities of Cardiff and Bristol which licences, quality-assures and supports the delivery of evidence-based public health promotion interventions. RC receives payment for this work.

MH has received payment for unrelated activity from Gilead Sciences Inc, Bristol Myers-Squibb and Jannsen, UK.

Contribution of authors:

GJM was responsible for recruitment, acquisition, analysis and interpretation of data and wrote the first draft of the manuscript. MH contributed to interpretation of data and finalisation of the manuscript. RC was responsible for conceptualisation of the study and contributed to interpretation of data and finalisation of the manuscript.

Keywords:

Alcohol drinking, qualitative, adolescent, social norms, peers

Data availability:

No additional data available.

For peer review only

Introduction

Although downward trends in alcohol use have been reported in the US and Europe,(1, 2) the reasons for this decline are as yet unclear, and while evidence is scant, studies indicate that the decline may not be uniform across all socio-demographic or levels of alcohol consumption.(3) Despite declines in the UK, recent figures show that 24% of 15-year olds in the UK have had a drink in the last week,(4) and by the age of 17, half of girls, and nearly two-thirds of boys, report drinking every week.(5) Adolescent drinking in the UK therefore remains a public health concern. Guidance from the Chief Medical Officer for England states that an alcohol-free childhood is the healthiest and best option, and if children do drink alcohol, it should not be until at least the age of 15 years; while if those aged 15-17 do drink, they should do so infrequently and on no more than one day per week.(6)

The critical role of both peer and parental influences in relation to adolescent alcohol consumption is well documented. Quantitative and qualitative studies have highlighted the impact of factors including parental monitoring, attitudes, communication and parental provision of alcohol via direct and indirect mechanisms.(7-12) In addition, peer influences, peer selection, the nature and reciprocity of friendships, and the social context of drinking, together generate peer group-based behavioural drivers of alcohol consumption,(13-21) and both descriptive and injunctive norms have been shown to be positively associated with alcohol use .(22, 23) Exposure to friends' alcohol-related content via social media has also been associated with alcohol use and later heavy episodic drinking, via the development of favourable peer injunctive norms, (24) demonstrating the links between use of social media, norms and social influences.

For peer review only - http://bmjopen.bmj.com/site/about/guidelines.xhtml

Normative social influence describes the social and emotional motivation to comply with the majority and the expectations of others to gain social approval and/or to avoid ridicule and social rejection, particularly in public settings where lack of conformity may be more evident (25, 26). Perceptions of the consensus view in a group, or collective norms, influence conformity, since the more consensual the group, the more isolated the 'deviant', and the more powerful the group in shaping the social space. In this way, alcohol use behaviour can be influenced by perceived descriptive norms (the perceived prevalence of alcohol consumption) and injunctive norms (the perceived approval of alcohol use) among young people and both have been shown to influence adolescent alcohol use at the individual and group level (27, 28))

While qualitative studies have reported the effect of peer, parental and wider cultural influences on young people's drinking, interaction and engagement with Social Networking Sites (SNS), and the integral nature of the social context,(13-15, 29-32) few recent qualitative studies have involved an in-depth examination of social and cultural influences on the initiation and escalation of alcohol use during early- to mid-adolescence in England, and the interaction between such behavioural influences. We sought to obtain data on social influences that would be applicable to the national context to inform development of preventive interventions. In addition, since little data are available to help to explain downward trends in alcohol use among young people in England,(2, 3, 33) it also remains important to gain a contemporary understanding of the impact of peers, social influences and cultural factors to offer insights around factors that might affect such trends.

In this paper, we report findings of a qualitative study that used theories of social influences and social norms as the theoretical lens through which to explore the social, cultural and behavioural drivers of alcohol consumption and the nature of drinking culture in mid-adolescence. Our exploration of the social worlds and drinking cultures of younger adolescents, and influences on

For peer review only - http://bmjopen.bmj.com/site/about/guidelines.xhtml

drinking practice, aims to inform the development of interventions to prevent excessive alcohol consumption and related harm.

For peer review only

For peer review only - http://bmjopen.bmj.com/site/about/guidelines.xhtml

Methods

While the initial aim of the study was to investigate the role of friendships in relation to drinking behaviour, we report here our findings regarding this and a wider range of determinants of behaviour, which reflects additional topics pertinent to young people that were raised by them and discussed in the interviews and which thereby feature in the data analysis.

Sampling and Recruitment

Our study sample included thirty young people aged 14-15 years who were recruited from four secondary schools in the South West of England. No participants dropped out of the study. The schools were in urban (n=3) and suburban/rural (n=1) areas and were in areas with diverse levels of deprivation as reflected by the index of multiple deprivation score for the ward. Students were randomly selected from the year group and snowball sampling was employed subsequently to recruit additional participants. A further twelve participants aged 14-18 years were recruited from youth groups, via leaders of the youth group, to ensure diversity in the sample in relation to socio-demographic characteristics and to enable data collection outside of the school environment. Leaders of youth groups disseminated information about the study and study materials and arranged interviews or the focus group. The sample included individuals who had, and hadn't, consumed alcohol. Parental consent and participant assent were given prior to participation for those aged under 16. Participants aged over 16 years gave informed consent. Recruitment took place between January and October 2017. The number of participants recruited was determined by the point at which saturation was reached i.e. when no new themes or perspectives were emerging in the interviews.

Data collection

A total of 36 semi-structured interviews were conducted (15 males, 21 females), three of which were paired interviews, and one of which was a triad interview, conducted with friends. Interviews were

For peer review only - http://bmjopen.bmj.com/site/about/guidelines.xhtml

conducted in meeting rooms in schools (n=30) and youth groups (n=6). One focus group (n=6 males) was also conducted in a youth group. Interviews were conducted by GJM (PhD), a female researcher with training in qualitative research. No relationship was established with study participants before commencement of data collection. Before starting the interview, participants were aware of the reasons for doing the research and the interviewer's aim of developing a preventive intervention in the future. Interviews were facilitated by a topic guide, which was used flexibly so that participants could explore views and opinions which were meaningful to them. The topic guide included questions around attitudes and perspectives around drinking, personal histories of alcohol use, risks and consequences of drinking, the role and influence of friendship groups and social networks, and family-related factors. Views on alcohol-related education and alcohol-related interventions were discussed but will be reported elsewhere. The participant sample included those who consumed alcohol and those who abstained, but many of those who chose not to drink alcohol discussed their views around alcohol use and perceptions and experiences relating to peers who did consume alcohol. Drinking patterns of participants are noted alongside quotes, based on comments made by participants during interviews. Interviews lasted on average 39 minutes (range 19-66 minutes) and were audio-recorded and transcribed verbatim. Interview data were anonymised. Participants received a £15 gift voucher for taking part.

Data analysis

Transcripts of interviews were imported into NVivo version 10 (QSR International, Brisbane) and analysed thematically using this software by GJM. Participants did not check or correct transcripts. Analysis aimed to capture emergent concepts and thus took an inductive approach. Transcripts were read and re-read and open line-by-line coding was conducted with a focus on understanding and capturing participants' experiences, behaviours, feelings, attitudes, and perceptions around alcohol use. We sought to explore relationships and patterns both within and between accounts. Different accounts were compared and contrasted, such that codes were progressively refined, and

For peer review only

connections mapped, to characterise relationships and identify underlying concepts and then categories and themes. Ongoing engagement with the data ensured that emerging concepts were grounded in participants' accounts. Throughout the process the researcher (GJM) wrote analytic memos and notes to capture thoughts around meanings, themes and relationships between themes. Emerging themes and concepts, and the theoretical basis for analysis, was discussed with the last author. Thematic patterns were considered within and across cases and between groups of cases e.g. those who did, or did not, drink alcohol.

Sociological theories relating to social influences, group identity and social norms were explored during analysis to enhance understanding around the theoretical basis of influences on the initiation and maintenance of alcohol use behaviour evident from the data.(25, 34, 35) Thus, these theories were used as a theoretical lens, which helped to explain the data. We also explored whether social practice theory (36-38) might help to explain alcohol use behaviour as a collective social practice during adolescence. However, while a social practice perspective helped to explain the social context of alcohol use and the meanings associated with alcohol consumption, it did not fully explain data regarding peer influences and/or pressures, which were key themes in our study. We also note that we did not specifically seek to examine the influence of social media in initial interviews, rather, the role and importance of social media emerged as a theme and was therefore explored in relation to social influences during data analysis.

Ethical considerations

Ethical approval for the study was obtained from the University of Bristol Faculty of Health Sciences Ethics Committee (study 131443 (8201)/2306).

For peer review only

Public involvement

We did not involve young people in this study directly, however, the authors engaged with young people advisory groups (YPAGs) prior to commencing the programme of research in which this qualitative study was embedded. The overall aim of the programme of research was to develop an intervention to reduce excessive alcohol use and harm among young people and this qualitative study, and engagement of YPAGs aimed to inform the design and theoretical basis of an preventive intervention.

For peer review only

Results

The major themes that emerged from the data were: normalisation and the wider culture, motivations, peer influence, pressure among peers, social media, and young people's drinking culture. Family influences were also a major theme, but we have not addressed them here, as we have previously reported similar findings elsewhere.(10)

Normalisation of drinking and the wider alcohol culture

Young people described alcohol consumption as a normalised practice and an accepted part of the cultural context. Young people accepted drinking as part of life and stressed the importance of autonomy around people's decisions regarding alcohol consumption. Exposure to teen films and social media presented alcohol consumption as fun and 'cool' and a means of enhancing popularity, while lacking details of negative consequences. Young people therefore frequently came into adolescence with clear pre-formed positive attitudes towards drinking and an understanding of its meanings in the social world (Supplementary material S1, quotes 1 and 2).

> *So it's just like you just grow up with like people around you like just drinking casually like with meals and stuff so no-one sees it as particularly dangerous … until like something actually goes wrong. Yeah. So you're kind of shielded from the actual dangers… (ID 17, F, drinker)*

Reflecting the assimilation of wider cultural norms, perceptions of what constituted reasonable alcohol use involved levels of consumption far above levels at which drinking would become hazardous or harmful (see also S1, quote 3).

> *INT: And how much do you think is ok would you say?*
>
> *RES: Not getting drunk like really drunk like you can't even walk (ID 29, M, non-drinker)*

For peer review only - http://bmjopen.bmj.com/site/about/guidelines.xhtml

Despite such misperceptions, participants (including those who did and did not drink alcohol) were aware of the balance between consuming alcohol for enjoyment and negative consequences, evidencing a clear awareness of the potential for a range of negative consequences to ensue if limits were overstepped, rendering the activity pointless or creating difficulties for others (S1, quotes 4 and 6). In addition, those who abstained, while noting acceptance of alcohol use within social groups, highlighted avoidance of such consequences through the absence of drinking in their own world.

> *I kind of fear the consequences of me drinking. And I also think about my family: if they were to find out they would just be like angry and annoyed at me (ID 16, M, non-drinker)*

Motivations to drink and the initiation of alcohol use

Drinking became an integral practice in young people's social worlds by age 14-16. Young people most frequently described a shared meaning of alcohol consumption relating to collective fun, enjoyment and being cool. Drinking also improved confidence, popularity, and engagement with friends while enabling young people to rebel, make memories, and experiment, while providing a means of managing mental illness, avoiding reality, excusing behaviour or escaping stress (S2, quotes 1 and 2).

Satisfying the range of motivations to drink was straightforward since alcohol was both readily available and cheap. Some described initiation of drinking among peer groups via an influential individual, who was the first to provide or drink alcohol (often via parents), setting an expectation of drinking at subsequent parties.

> *RES: I think first party was in October when it was like, oh there's going to be alcohol there. .. And that was sort of an initiation. [LAUGHS] I don't know.*
>
> *INT: Yeah.*
>
> *RES: But it's sort of like the – yes it's after that party then it's sort of expected*

that there is at other ones, maybe. (ID 8, F, non-drinker)

Young people's drinking and party culture

Drinking practice was rooted in house parties, which were viewed as a safer and more controlled setting than were outdoor settings. Parties' meanings were centred around the inherently social nature of the events, providing opportunities for socialising, shared experiences and a sense of togetherness with friends, as well as opportunity for drunkenness, which was a widely accepted norm. For many, alcohol made parties more enjoyable (S3, quote 1) and getting drunk provided an aim and purpose, while providing the means of looking popular, 'hard', or cool. Thus, widely held understandings, or aims, were to drink and 'get smashed' (S3, quotes 2 and 3), with the pace and extent of drunkenness often being a source of direct or indirect competition. Participants also described indirect pressures involving stigmatising insults linked to competence around intoxication.

RES: ..it's kind of like who can drink more and not get drunk, but it's like not a competition in that way, yeah.

INT: So it's not a competition?

RES: No, it's not a competition, but it feels like that because after you're a bit, oh, this guy's a lightweight, and that. (ID 37, M, non-drinker)

Thus, it was important to tread a line between appearing competent in tolerating alcohol to avoid stigmatising labelling as a 'lightweight' (S3, quote 4), but to remain in control to avoid ruining a party and/or require care from friends, which was associated with reputational risk and disgruntlement from friends. Those who often took on the role of looking after friends described feelings of reluctance and resentment around missing out on enjoyment, or concern around managing the effects of intoxication.

The first feeling is panic and then you feel just disappointed and you're just angry. You feel… I feel a little bit angry but especially if they've done it before you know

For peer review only

what you've done, you know how like what your limit is and yet you go out of your way to do it.. (ID 15, F, moderate drinker)

The disparity between individual autonomy in decisions around intoxication and the requirement for help from others thereafter was also viewed negatively (S3, quote 5).

Social influences on adolescent alcohol use

1. Peer influence

Social influences became paramount in mid-adolescence, with young people frequently reporting a desire to 'fit in' with friends. Those who drank earlier and to a greater extent acted as influential 'role models' for drinking (S4.1, quote 1). Thus, young people implicitly understood the social significance of joining in and facilitating belonging in the social world to enhance social status or to avoid social sanctions.

Kind of like 'cause everyone else is around you drinking, you kind of feel like you have to otherwise you don't really fit in um so that's what kind of happens to me most of the time. (ID 27, F, drinker)

Social influences also facilitated a rise in the prevalence of drinkers, thus enhancing proximal social influences and further contributing to the normalisation of alcohol consumption and the feedback cycle of conformity, with those who abstained thus feeling more isolated and dependent on non-drinking friends to diminish felt pressures.

Social influence led to perceived requirements to join in in some way, and those who didn't join in with the vibe of the party were a 'buzz kill' (S4.1, quote 2). As such, a somewhat circular effect sustained the party culture, whereby the increasing prevalence of drinking strengthened group norms and social learning processes that generated a desire to drink to feel included, and which generated a social environment in which abstaining could be alien (see also S4.1, quote 3).

For peer review only - http://bmjopen.bmj.com/site/about/guidelines.xhtml

I didn't intend to drinking alcohol last year … but it just kind of happens, like when you're invited to a party you go just go and there's alcohol and you get, you get told there's going to be alcohol, and you just drink it to have a good time anyway 'cause everyone else is. (ID 39, M, previous, now infrequent drinker)

Those who didn't comply with norms described feelings of awkwardness, embarrassment or boredom, or they avoided parties altogether, while others were incentivised to drink to avoid having to look after intoxicated friends (S4.1, quotes 4-6).

Notably, however, peer influences could have counter-effects, such that participants described alcohol consumption as pointless, expressing confusion around friends' drinking practices, while others reported aversion to role models of intoxication, which could strengthen views around abstinence (see also S4.1, quote 7).

I wouldn't do it because I have seen what in the middle, I have seen people feeling sick, all in the bath, like going on the street, I was like no way I am not doing that, you see what the impacts are if you drink. You see what would happen and I don't want that at all. (ID 6, F, non-drinker)

Such individuals were less compliant, or non-compliant, with the group norm and often played a greater 'mum' role in supporting and helping others within the social group (S4.1, quote 8). Those who were non-compliant, acted in keeping with family values and messages, and were unaffected by group norms.

I don't know what, what my mum would think if I become a drinker, so I – and I think she's kind of built it in me that it's, it's now in my moral code not to drink.(ID 12, M, non-drinker)

For peer review only - http://bmjopen.bmj.com/site/about/guidelines.xhtml

Individuals who abstained from drinking described the importance of friends with similar attitudes and behaviours around drinking and there was some evidence for peer selection in this group. Such friends provided support for their viewpoint and provided some protection from felt pressure or exclusion, enabling them to abstain and/or to enjoy social events more.

> *..if I was on my own it would have been worse, because you're just looking around and everyone's drinking. Not that it would lead me to drinking, but it's still better that I had friends who just don't do that at all: it made it much more fun (ID 16, M, non-drinker)*

Those who abstained frequently described strong interests in extra-curricular activities, particularly sports, a strong religious faith, and/or clear core values and aligned family viewpoints (which highlighted strong, clearly stated disapproval for alcohol use), which altered the meaning of alcohol use and reduced, or counteracted, the effects of social influences and norms (S4.1, quote 9). In addition, some of those who abstained reported family history of awareness of dependence or alcohol-related problems, which increased concern around the risks and negative consequences of alcohol consumption.

> *I'm pretty sure I'm glad not to like I don't want to start doing it because then maybe like later life I will be addicted and I don't want to be. (ID20, M, non-drinker)*

2. The influence of social media

The use of social media was integral to adolescent drinking behaviour and practice, particularly use of Instagram or the multimedia messaging app Snapchat. Posting pictures and videos from parties reflected the meanings associated with alcohol use, thus portraying participants being cool and sociable, to enhance popularity and social status. The ubiquity and prominence of social media weaved into young people's lives a relatively consistent, positive view of alcohol consumption that

For peer review only - http://bmjopen.bmj.com/site/about/guidelines.xhtml

For peer review only

could act as another source of social influence and a visual depiction of norms that could contribute to a felt pressure to drink (see also S4.2, quotes 1 and 2).

> *Because in a friendship it's more like direct, like they're going to tell you that, but like, on a big scale like social media it kind of like tells you like that you should be drinking, like when you can, and the norm is to, and then I think you feel a bit pressured into it. (ID 37, M, non-drinker)*

Social media used to demonstrate social capital, drinking 'competence' and maturity. Some were described as sipping drinks just to look right in pictures, while others aimed to depict their prowess in consumption.

> *I've noticed that a lot in my year group and like they'll kind of just… even if they don't really want to drink it they'll kind of just hold it there and they'll just like sip and stuff so they can have it for photos. (ID 34, F, moderate drinker)*

Nevertheless, views on the impact on social media were mixed. A minority expressed caution about posting images of themselves drinking owing to potential reputational risk. A minority also reported a view that drinking was unaffected by social media, although it was nevertheless described as a key component of drinking practice.

The pressurised environment

The combination of social media, social influences and social learning, alongside the meanings associated with drinking (e.g. belonging, popularity) and wider cultural influences, created a pressurised environment around drinking (Figure 1). Thus, the very interest in fitting in or feeling a need to join in with friends was felt to be subtly pressurising, while being the only person not drinking felt awkward and created unease, further contributing to a felt pressure.

For peer review only - http://bmjopen.bmj.com/site/about/guidelines.xhtml

Because you see everyone's doing it then you felt .. er like obliged to do it as well sometimes. Um I don't often, like, myself but you can definitely tell other people um it's like, oh everybody else is doing it so then I have to do it as well to fit in, it's like one of those sorts of things and also if your friends are drunk then it sort of becomes a thing where oh do you want mine you can have some, have some, and then you're like oh my gosh like do I accept, do I not (ID 15, F, moderate drinker)

Branding of individuals as 'wimps' or awarding 'lad points' for intoxication associated with courage and humour, further created incentives and obligations to drink to excess (S5, quote 1). The inextricable link between drinking alcohol and socialising meant that resisting pressures or challenging friends risked losing credibility as an integral and fun group member, or risked jeopardising friendships (see also S5, quotes 2 and 3).

INT: .. and how does that make you feel if you are saying no?

RES: Um if you're like receiving yeah then you can kind of feel like it makes you not want to be in that situation, it makes you just like feel oh I don't feel safe in this environment anymore cos now I'm doing something that I don't want to do and you're just like what do I do, I can't deal with this situation and you don't want to not be friends with people who you're friends with, you don't want to break friendships over it but it's one of those things where you kind of if you don't like it then you have to say no and if your friends didn't accept that then it's like you have to sort that out with your friends yourself, that sort of thing. (ID 15, F, moderate drinker)

Pressure to drink was felt in a range of ways, including subtle and indirect pressure through offers of alcoholic drinks, the felt need to be fun and to fit in, or proximal social influence. However, participants also reported more direct pressure through goading or direct insults, leading some to

For peer review only

For peer review only - http://bmjopen.bmj.com/site/about/guidelines.xhtml

describe feeling ‘forced to drink’ and others wishing to avoid appearing cowardly. Overall, the social environment was felt to be conducive to feeling obliged to drink, or being ‘pushed in the direction’ towards alcohol use (see also S5 quotes 4 and 5).

> *.. I used to think alcohol was really bad but I guess as like it becomes more talked about, you're kind of dragged into it.*
>
> *INT: Mm. It’s interesting you say dragged into it.*
>
> *RES: Not dragged – I’d say like kind of um pushed in the direction. (ID 27, F, drinker)*

Nevertheless, such felt pressures were often unclear to the friends in question who were drinking, who therefore pressurised others unknowingly. Drinkers did not consider their actions to be pressurising, rather a gesture to enable or encourage friends to join in (see also S5, quote 6).

> *They just think they’re saying it does – it’s just a laugh like, if they’re – they’re kind of – they’re almost trying to make their experience more enjoyable by saying if you do this… .. it will make it better, but I don’t think that’s the same kind of response for the person on the other end. (ID 33, M, drinker)*

As outlined above, for non-drinkers, having friends with a similar viewpoint provided support and reduced felt pressure.

> *.. I don’t know if I didn’t have my friends or anyone to tell me not to drink, I’d probably be drinking by now. .. So, I’m kind of happy that they’re around. (ID 12, M, non-drinker)*

For peer review only

Discussion

In this paper, we have demonstrated how the wider alcohol culture normalised alcohol use for young people, helping to generate perceptions of drinking as a social practice associated with being cool, mature and popular as well as an important means of enhancing enjoyment and social status, in support of evidence regarding primary social and enhancement drinking motives in young adults (39). The ubiquity of alcohol use generated normative social influences and opportunities for social learning around initiation and continuation of alcohol use. Together with embedded meanings and expectancies, the interaction between peer norms, social influences and the effects of social media generated a pressurised environment in which young people felt obliged to comply and join in.

The strengths of our study include the gender-balanced sample of young people from both schools and youth groups, including those who used alcohol and those who abstained. We also used one-to-one interviews, which provided space for young people to share their views around drinking, without concerns around disagreement or judgement from peers or friends. A small proportion of interviews were conducted with dyads with one triad, however, we did not find evidence that participants exaggerated or withheld views and perspectives in these pairs, and paired interviews were conducted when expressed as a preference by the participants. Nevertheless, our data were not triangulated, and participants were recruited from urban and suburban settings only, so our findings cannot necessarily be generalised to different geographical or demographic contexts. Interestingly, although parental messages and the off-putting effect of observing intoxicated role models were described by some moderate drinkers, our data did not provide evidence regarding factors that might be affecting downward trends in the prevalence of alcohol use among young people in England, and further qualitative and quantitative research will be needed to explore such trends. Lastly, while we have reported that social influences and pressures are a critical influence on young people's alcohol use, further research will be needed to explore exactly how such influences could be effectively addressed in public health interventions for young people.

For peer review only - http://bmjopen.bmj.com/site/about/guidelines.xhtml

For peer review only

The frequently reported desire to fit in with the group, and unease expressed in relation to abstinence, highlighted the central role of normative social influence in generating a collective practice in which intoxication was accepted and expected. Evidence suggests that both direct and indirect pathways of peer influences and pressures may exist, which can contribute to generating social influences (and can act together with peer selection (19, 40)). Pathways can be direct, including offers of drinks, dares, or goading, (fitting with social and enhancement motives for drinking)(41) and indirect, including modelling of peer drinking and/or contributions to the formation of norms, beliefs and expectancies,(42, 43) (fitting with conformity motives for drinking)(39) which were also evident in our study. In addition, studies have reported an impact of being around peers who drink alcohol, either through proximity to social acquaintances who consume alcohol or through time spent with drinking peers,(44, 45) highlighting the important impact of the rise in prevalence of alcohol use through adolescence. This was evident in our study since being around other drinkers and being immersed in a practice in which drinking was highly prevalent (contributing to descriptive norms) contributed to the felt obligation to drink.

Frequent reports by young people of needing to 'fit in' suggest that injunctive norms, or perceptions of peer approval of drinking, were also critical. Thus, our findings support those of quantitative studies, which have reported associations between both descriptive and injunctive norms and frequency and quantity of adolescence alcohol use and heavy episodic drinking,(22, 46). Specifically, one study reported a greater impact of injunctive compared to descriptive norms, with the former associated with a range of measures of alcohol use as well as alcohol-related negative consequences.(22)

In addition to social norms and influences among peers, social media, or social networking sites (SNS), played a role in providing a physical depiction of positive alcohol-related descriptive and

For peer review only - http://bmjopen.bmj.com/site/about/guidelines.xhtml

injunctive norms, which was an integral part of the social practice of adolescent alcohol use. Thus, we have reported evidence to support a previous study describing ‘intoxigenic digital identities’ and ‘intoxigenic digital spaces’ that contribute to the normalisation of alcohol use.(47) Similarly, a qualitative study of young women’s drinking cultures described the critical nature of SNS in facilitating displays of popularity, belonging, membership of the social network and ‘friendship fun’.(32) Such media also bring about opportunities for misperceptions owing to the circulation of positive imagery, which can be interpreted at face value or as ‘truth’.(48, 49) Quantitative data have demonstrated an association between exposure to friends’ alcohol-related SNS content and initiation of alcohol use and stronger pro-alcohol peer injunctive norms, highlighting how the provision of opportunities for observation, comparison and role-modelling of behaviours through SNS can impact on norm perception and behaviour.(24) In addition, others have reported an association between alcohol promotion through digital media and adolescent alcohol consumption.(50, 51)

Taken together, therefore, evidence suggests the need for public health interventions to effectively address both peer norms and social influences during adolescence, including the effects of SNS, to enhance the likelihood of effective prevention. Furthermore, the evidence of felt pressure resulting from the influences discussed above suggests that many young people feel a lack of autonomy around alcohol consumption, and constraint around behavioural decisions within their social groups. Thus, in addition to addressing norm perception and normative social influence, there is a need for interventions to find ways of overcoming such constraints and fostering greater resistance to conformity. Indeed our sample included participants who reported abstaining from alcohol use, and navigation through the social world resisting influences of drinking peers, with critical support from non-drinking friends, the importance of which has been reported by others (52). Thus, fostering greater resistance to conformity may require a strengthening of the voice and influence of those in the minority group (i.e. those who abstain from drinking) as outlined in Moscovici’s theory of

For peer review only - http://bmjopen.bmj.com/site/about/guidelines.xhtml

minority influence (53, 54), which may be increasingly feasible in light of reported downward trends in adolescent alcohol consumption (3) and thus an increase in the proportion of non-drinking peers. Moscovici also highlights how influence goes beyond shaping people to a system, but that it continually changes a social system, thus the influence of increasing proportions of abstainers, who in our study reported counter-effects of peer influence and aversion to role models of intoxication, may increasingly shift alcohol use downwards at a population level (25) as the benefits of the 'deviant' or non-conforming behaviour or perspective become evident. It has also been suggested that feelings of powerlessness during adolescence may be overcome through enhanced social status for young people, for instance through opportunities for creative activities and active engagement in communities.(55)

Conclusions

Our study has demonstrated the effect of the interplay between cultural norms, social influences, social pressures, and social media, particularly the critical role of normative social influence, in driving the escalation and normalisation of alcohol use in mid-adolescence. Our findings suggest that there is a need for a combination of approaches to effectively prevent excessive alcohol use among young people. Interventions in young people need to target normative social influence and peer norms to interrupt the rise in prevalence of excessive drinking, while aiming to enhance social status and the ability to challenge social pressures, to increase the likelihood of preventing alcohol-related harm.

For peer review only - http://bmjopen.bmj.com/site/about/guidelines.xhtml

Figure 1.

Intersection of social and cultural influences on adolescent alcohol use and generation of a pressurised environment.

Note that family influences have been addressed elsewhere.

For peer review only

For peer review only - http://bmjopen.bmj.com/site/about/guidelines.xhtml

Acknowledgements

GJM was supported by an NIHR post-doctoral fellowship award (PDF-2013-06-026). This report is independent research supported by the National Institute for Health Research (Post-Doctoral Fellowship, PDF-2013-06-026). The views expressed in this publication are those of the author(s) and not necessarily those of the NHS, the National Institute for Health Research or the Department of Health and Social Care.

The work was undertaken with the support of The Centre for the Development and Evaluation of Complex Interventions for Public Health Improvement (DECIPHer), a UKCRC Public Health Research Centre of Excellence. Joint funding (MR/KO232331/1) from the British Heart Foundation, Cancer Research UK, Economic and Social Research Council, Medical Research Council, the Welsh Government and the Wellcome Trust, under the auspices of the UK Clinical Research Collaboration, is gratefully acknowledged.

RC is supported by the University of Bristol.

We are extremely grateful to the schools, youth groups and participants for their assistance and participation in this study. We would also like to thank Bristol Drugs Project for their assistance with our work.

For peer review only

For peer review only - http://bmjopen.bmj.com/site/about/guidelines.xhtml

References

1. Looze Md, Raaijmakers Q, Bogt Tt, Bendtsen P, Farhat T, Ferreira M, et al. Decreases in adolescent weekly alcohol use in Europe and North America: evidence from 28 countries from 2002 to 2010. European Journal of Public Health. 2015;25(suppl_2):69-72.
2. Kraus L, Seitz, N-N, Piontek D, Molinaro S et al,. "Are The Times A-Changin'"? Trends in Adolescent Substance Use in Europe. Addiction. 2018.
3. Pape H, Rossow I, Brunborg Geir S. Adolescents drink less: How, who and why? A review of the recent research literature. Drug and Alcohol Review. 2018;37(S1):S98-S114.
4. NHS Digital. Smoking, drinking and drug use among young people. England 2016. UK.: National Statistics; 2017.
5. Public Health England. Data intelligence summary: Alcohol consumption and harm among under 18 year olds. UK.: Public Health England.; 2016. Contract No.: Publications gateway number: 2016173.
6. Department of Health. Guidance on the consumption of alcohol by children and young people. Report from Sir Liam Donaldson, Chief Medical Officer for England. London: Department of Health; 2009.
7. Rossow I, Keating P, Felix L, McCambridge J. Does parental drinking influence children's drinking? A systematic review of prospective cohort studies. Addiction. 2016;111(2):204-17.
8. Mattick RP, Clare, Philip J., Aiken, Alexandra, Wadolowski, Monika, Hutchinson, Delyse, Najman, Jackob, Slade, Tim, Bruno, Raimondo, McBride, Nyanda, Kypri, Kypros, Vogl, Laura, Degenhardt, Louisa. Association of parental supply of alcohol with adolescent drinking, alcohol-related harms, and alcohol use disorder symptoms: a prospective cohort study. The Lancet Public Health. 2018;3(2):e64-e71.
9. Özdemir M, Koutakis N. Does promoting parents' negative attitudes to underage drinking reduce adolescents' drinking? The mediating process and moderators of the effects of the Örebro Prevention Programme. Addiction. 2016;111(2):263-71.
10. Jacob N, MacArthur GJ, Hickman M, Campbell R. A qualitative investigation of the role of the family in structuring young people's alcohol use. The European Journal of Public Health. 2015.
11. Campbell JM, Oei TP. A cognitive model for the intergenerational transference of alcohol use behavior. Addictive Behaviors. 2010;35(2):73-83.
12. Mahedy L, MacArthur, GJ, Hammerton, G, Edwards, AC, Kendler, KS, Macleod, J, Hickman, M, Moore, SC, Heron J. The effect of parental drinking on alcohol use in young adults: the mediating role of parental monitoring and peer deviance. Addiction.epub.:doi: 10.1111/add.14280.
13. MacArthur GJ, Jacob N, Pound P, Hickman M, Campbell R. Among friends: a qualitative exploration of the role of peers in young people's alcohol use using Bourdieu's concepts of habitus, field and capital. Sociology of Health & Illness. 2016:n/a-n/a.
14. de Visser RO, Wheeler Z, Abraham C, Smith JA. 'Drinking is our modern way of bonding': Young people's beliefs about interventions to encourage moderate drinking. Psychology & Health. 2013;28(12):1460-80.
15. Niland P, Lyons AC, Goodwin I, Hutton F. "Everyone can loosen up and get a bit of a buzz on": Young adults, alcohol and friendship practices. International Journal of Drug Policy. 2013;24(6):530-7.
16. Kelly AB, Chan GCK, Toumbourou JW, O'Flaherty M, Homel R, Patton GC, et al. Very young adolescents and alcohol: evidence of a unique susceptibility to peer alcohol use. Addictive Behaviors. 2012;37(4):414-9.
17. Ali MM, Dwyer DS. Social network effects in alcohol consumption among adolescents. Addictive behaviors. 2010;35:337-42.

For peer review only - http://bmjopen.bmj.com/site/about/guidelines.xhtml

18. Percy A, Wilson, J., McCartan,C., McCrystal, P. Teenage drinking cultures. York: Joseph Rowntree Foundation; 2011.
19. Leung RK, Toumbourou JW, Hemphill SA. The effect of peer influence and selection processes on adolescent alcohol use: a systematic review of longitudinal studies. Health Psychology Review. 2014;8(4):426-57.
20. Fujimoto K, Valente TW. Decomposing the components of friendship and friends' influence on adolescent drinking and smoking. The Journal of adolescent health : official publication of the Society for Adolescent Medicine. 2012;51:136-43.
21. Bot SM, Engels RCME, Knibbe Ra, Meeus WHJ. Friend's drinking behaviour and adolescent alcohol consumption: the moderating role of friendship characteristics. Addictive behaviors. 2005;30:929-47.
22. Pedersen ER, Osilla KC, Miles JNV, Tucker JS, Ewing BA, Shih RA, et al. The role of perceived injunctive alcohol norms in adolescent drinking behavior. Addictive Behaviors. 2017;67:1-7.
23. Teunissen HA, Kuntsche E, Scholte RHJ, Spijkerman R, Prinstein MJ, Engels RCME. Friends' drinking norms and male adolescents' alcohol consumption: The moderating role of performance-based peer influence susceptibility. Journal of Adolescence. 2016;53:45-54.
24. Nesi J, Rothenberg WA, Hussong AM, Jackson KM. Friends' Alcohol-Related Social Networking Site Activity Predicts Escalations in Adolescent Drinking: Mediation by Peer Norms. Journal of Adolescent Health.
25. Turner JC. Social Influence. Buckingham: Open University Press; 1991.
26. Asch SE. Studies of independence and conformity: A minority of one against a unanimous majority. . Psychological Monographs: General and Applied. 1956;70:1-70.
27. François A, Johnson SL, Waasdorp TE, Parker EM, Bradshaw CP. Associations Between Adolescents' Perceptions of Alcohol Norms and Alcohol Behaviors: Incorporating Within-School Variability. American journal of health education. 2017;48(2):80-9.
28. Amialchuk A, Ajilore O, Egan K. The influence of misperceptions about social norms on substance use among school-aged adolescents. Health Economics. 2019;28(6):736-47.
29. Townshend TG. Youth, alcohol and place-based leisure behaviours: A study of two locations in England. Social Science & Medicine. 2013;91(0):153-61.
30. Bremner P, Burnett, J., Nunney, F., Ravat, M., Mistral, W. Young people, alcohol and influences. A study of young people and their relationship with alcohol. York; 2011.
31. Scott S, Shucksmith J, Baker R, Kaner E. 'Hidden Habitus': A Qualitative Study of Socio-Ecological Influences on Drinking Practices and Social Identity in Mid-Adolescence. International Journal of Environmental Research and Public Health. 2017;14(6):611.
32. Atkinson AM, Sumnall HR. 'If I don't look good, it just doesn't go up': A qualitative study of young women's drinking cultures and practices on Social Network Sites. International Journal of Drug Policy. 2016;38:50-62.
33. Larm P, Livingston, M., Svensson, J., Leifman, H., Raninen, J. The increased trend of non-drinking in adolescence: The role of parental monitoring and attitudes toward offspring drinking. Drug and Alcohol Review. 2018;37(S1):S34-S41.
34. Tajfel H, Flament, C., Billig, M.G. et al,. Social categorisation and intergroup behaviour. European Journal of Social Psychology. 1971;5:245-53.
35. Sherif M. The psychology of social norms. New York.: Harper and Brothers; 1936.
36. Blue S, Shove E, Carmona C, Kelly MP. Theories of practice and public health: understanding (un)healthy practices. Critical Public Health. 2016;26(1):36-50.
37. Shove E. PM, Watson M.,. The Dynamics of Social Practice: Everyday Life and How it Changes. London: Sage; 2012.
38. Meier PS, Warde A, Holmes J. All drinking is not equal: how a social practice theory lens could enhance public health research on alcohol and other health behaviours. Addiction. 2018;113(2):206-13.

For peer review only - http://bmjopen.bmj.com/site/about/guidelines.xhtml

39. Mackinnon SP, Couture M-E, Cooper ML, Kuntsche E, O'Connor RM, Stewart SH, et al. Cross-cultural comparisons of drinking motives in 10 countries: Data from the DRINC project. Drug and Alcohol Review. 2017;36(6):721-30.
40. Osgood DW, Ragan DT, Wallace L, Gest SD, Feinberg ME, Moody J. Peers and the Emergence of Alcohol Use: Influence and Selection Processes in Adolescent Friendship Networks. Journal of Research on Adolescence. 2013;23(3):500-12.
41. Kuntsche E, Knibbe R, Gmel G, Engels R. Why do young people drink? A review of drinking motives. Clinical Psychology Review. 2005;25(7):841-61.
42. Borsari B, Carey KB. Peer influences on college drinking: A review of the research. Journal of Substance Abuse. 2001;13(4):391-424.
43. Studer J, Baggio S, Deline S, N'Goran AA, Henchoz Y, Mohler-Kuo M, et al. Peer pressure and alcohol use in young men: A mediation analysis of drinking motives. International Journal of Drug Policy. 2014;25(4):700-8.
44. Dallas R, Field M, Jones A, Christiansen P, Rose A, Robinson E. Influenced but Unaware: Social Influence on Alcohol Drinking Among Social Acquaintances. Alcoholism: Clinical and Experimental Research. 2014;38(5):1448-53.
45. Salvy S-J, Pedersen ER, Miles JNV, Tucker JS, D'Amico EJ. Proximal and distal social influence on alcohol consumption and marijuana use among middle school adolescents. Drug and Alcohol Dependence. 2014;144:93-101.
46. Song E-Y, Smiler AP, Wagoner KG, Wolfson M. Everyone Says It's OK: Adolescents' Perceptions of Peer, Parent, and Community Alcohol Norms, Alcohol Consumption, and Alcohol-Related Consequences. Substance Use & Misuse. 2012;47(1):86-98.
47. Griffiths R, Casswell S. Intoxigenic digital spaces? Youth, social networking sites and alcohol marketing. Drug and Alcohol Review. 2010;29(5):525-30.
48. Moreno MA, Briner LR, Williams A, Brockman L, Walker L, Christakis DA. A Content Analysis of Displayed Alcohol References on a Social Networking Web Site. Journal of Adolescent Health.47(2):168-75.
49. McCreanor T, Lyons A, Griffin C, Goodwin I, Moewaka Barnes H, Hutton F. Youth drinking cultures, social networking and alcohol marketing: implications for public health. Critical Public Health. 2012;23(1):110-20.
50. Lobstein T, Landon J, Thornton N, Jernigan D. The commercial use of digital media to market alcohol products: a narrative review. Addiction. 2017;112:21-7.
51. Scott S, Muirhead C, Shucksmith J, Tyrrell R, Kaner E. Does Industry-Driven Alcohol Marketing Influence Adolescent Drinking Behaviour? A Systematic Review. Alcohol and Alcoholism. 2017;52(1):84-94.
52. Rees C, Wallace D. The myth of conformity: Adolescents and abstention from unhealthy drinking behaviors. Social Science & Medicine. 2014;108:34-45.
53. Moscovici S, Personnaz, B.,. Studies in Social Influence. V. Minority Influence and Conversion Behavior in a Perceptual Tast. Journal of Experimental Social Psychology. 1980;16:270-82.
54. Moscovici S, Lage E. Studies in social influence III: Majority versus minority influence in a group. European Journal of Social Psychology. 1976;6(2):149-74.
55. Pound P, Campbell R. Locating and applying sociological theories of risk-taking to develop public health interventions for adolescents. Health Sociology Review. 2015;24(1):64-80.

For peer review only

For peer review only - http://bmjopen.bmj.com/site/about/guidelines.xhtml

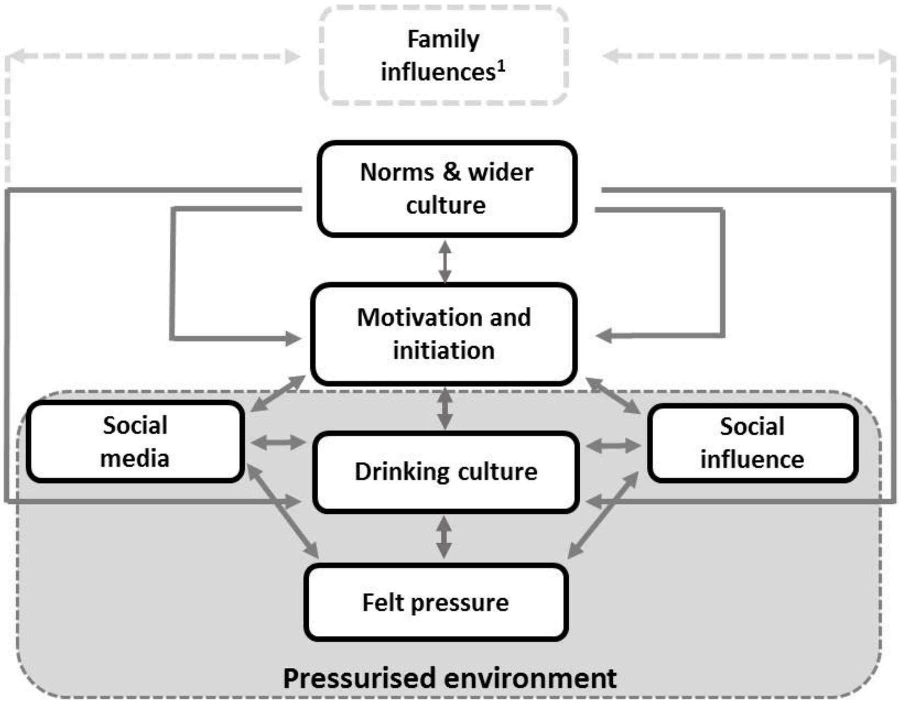

Figure 1. Intersection of social and cultural influences on adolescent alcohol use and generation of a pressurised environment.

[1] Family influences have been addressed elsewhere.

Intersection of social and cultural influences on adolescent alcohol use and generation of a pressurised environment. Note that family influences have been addressed elsewhere.

153x141mm (150 x 150 DPI)

Supplementary Material: Additional quotes

S1. Normalisation of drinking and the wider alcohol culture

1. *Yes but also like in films and like series, and social media, parties just look like crazy cus everyone's like getting drunk, so people who kind of their aim is, I wanna be cool, I wanna have lots of friends, and I wanna be the popular one. They think, the only way to do that is to go to the parties, cus that's what all the films say. (ID 13b, F, non-drinker)*

2. *I didn't really know that what it can actually do to you. I just thought oh it makes you happy and that's it, that's all you really hear about. (ID 21, F, drinker)*

3. *RES: … I think drinking [sic: in adults] is fine because like obviously people really enjoy like wine and stuff, um, but I think it's bad when you're like addicted or like drinking more than is necessary. Yeah.*

   INT: And how much would that be do you reckon, the drinking more than you need to?

   RES: Um, like, I dunno, if someone was drinking like a bottle of wine a day that's like… that's just like someone who's like… it's like kind of like a normal amount to drink but it's a lot. But if someone's actually drunk throughout the day that's terrible I think. Yeah. (ID 17, F, drinker)

4. *Well there's a point where it's like, not fun anymore and also, it's just not cool anymore. It can be seen as like, cool having a few cans, getting drunk but then like after that it's just silly. And people will like, ignore you, in that situation (ID 13b, F, non-drinker)*

For peer review only - http://bmjopen.bmj.com/site/about/guidelines.xhtml

5. *INT: Yeah, and what might be some of the bad things that you mention there?*

RES: Getting addicted or feeling sick the next day. You can do bad things when you're drunk.

INT: Bad things?

RES: Well you don't really know what you're doing. (ID 40, M, light drinker)

6. *INT: And when you say kind of light drinking can I ask a bit what that would be?*

RES: Maybe slightly tipsy but then like to the point where you can't walk is… there's n… there's no point.

INT: Yeah. Okay. So, up to that point it's…?

RES: Well, slightly tipsy, [inaudible] but then anything more is pretty pointless but… yeah.

INT: Yeah. And pointless because?

RES: It just… it's sort of taking the fun out of it and it's just… you're just being stupid and not controlling at all.

INT: Hmm. And what sort of things might happen at that point then?

RES: They'd hurt themselves.

INT: Okay. Yeah.

RES: Er, could hurt others maybe. Yeah. (ID 22, M, light drinker)

For peer review only

S2. Motivations to drink and the initiation of alcohol use

1. *.. you could forget your troubles maybe and just live in the moment and just go, 'I'll deal with the problems later. I'll have fun now'. (ID 22, M, light drinker)*

2. *I think it's a good social thing like it makes it easier for you to talk to people, you know, and you can - you don't have to worry about like making a fool of yourself because you have an excuse and stuff. (ID 18, F, drinker)*

For peer review only

For peer review only - http://bmjopen.bmj.com/site/about/guidelines.xhtml

S3: Young people's drinking and party culture

1. *People are saying oh its so, it will be so dead like without them drinking, like it will just be so boring and stuff, but they say like oh its better when we are drinking because it's more fun and like everybody is falling and everybody is like laughing. (ID 6, F, non-drinker)*

2. *Yeah, some people like – some people might usually go into like say the party and stuff and just say like "Oh I wanna get smashed" or something like that. (ID 36, M, drinker)*

3. *It's just mainly like parties, I guess, like people just think, it's a party, you have to drink, basically. (35b, F, non-drinker)*

4. *I'm not like – people often call you, um, a lightweight when you drink and get drunk, and it's like that's an offensive term. It's quite embarrassing if you get called that, but yeah, I think there's a lot of stigma towards that. (ID 32, F, drinker)*

5. *RES: I could be their friend and then they could do something stupid and I'd have to look after them or go and try and fix it, um, yeah.*

   INT: And how would that make you feel doing that?

   RES: Quite annoyed because they got themselves like this. (ID 22, M, light drinker)

For peer review only - http://bmjopen.bmj.com/site/about/guidelines.xhtml

S4. Social influences on adolescent alcohol use

4.1. Peer influence

1. *INT: And what do you think might have changed those people's opinion, how you said some people didn't and now do?*

   RES: Probably because they've seen the first people do it and once somebody…

   INT: Hmm.

   RES: … one person starts doing it and then the next person then I think eventually it becomes like, everyone just wants to try it and then if they like it then they'll do it again and again. (ID 34, F, moderate drinker)

2. *Like one party we were at there's a group of people and they were talking about revision and so you just like walk away from them, and you're like 'oh, they're such like a buzz-kill' because it's not… they don't have to be drunk but they're just like changing… they're like shifting the… like the, um, like the vibe of the party to more like, I dunno, not as funny. (ID 17, F, drinker)*

3. *INT: What do you think are the main reasons people might drink?*

   RES: Um, just they're eager to drink, and their friends are drinking. They will just drink. Like, it's... I mean, sometimes it's not even peer pressure; it's just seeing other people doing it, and you just want to do it as well, just to fit in. (ID 16, M, non-drinker)

4. *I know definitely that I felt a bit like not left out but like, not part of it, that's why I didn't enjoy it. It was really boring cus like I didn't wanna talk to anyone because*

For peer review only - http://bmjopen.bmj.com/site/about/guidelines.xhtml

they were all just like really drunk. So I just stayed like half an hour. (ID 13a, F, non-drinker)

5. *RES: Maybe like if you're invited to a party and everyone else there. You don't really wanna be the one that…the only one not.*

   INT: Um. How would it feel if you were the only person who wasn't do you think?

   RES: Embarrassed maybe.

   INT: Um and why do you think that is?

   RES: Um 'cause you don't feel like you fit in the group. (ID 40, M, light drinker)

6. *But I think it's more like yeah you don't want to be sober at a party if you don't want to be the person to look after all your friends.* (ID 21, F, drinker)

7. *Because they see people when they're drunk and they'll think well that… that may… may… you may think is fun but it really doesn't look that fun cos you're jumping about and jumping on your head and stuff like that. What's the point? (ID 22, M, light drinker)*

8. *RES 1: .. in my friendship group I'm kind of like the mum like I'm always like.*

   RES 2: Me too.

   RES 1: I'm always like just making sure everyone's ok.

   RES 2: Checking everyone's all right. (ID 31a F; 31b, F, moderate drinkers)

9. *I have had the invites from my friends but I've chosen not to go because I find that sport is more important to me, than just going drinking. (ID 37, M, non-drinker)*

For peer review only - http://bmjopen.bmj.com/site/about/guidelines.xhtml

4.2. The influence of social media

1. *They kind of make you feel like you want to drink more cus it's like they're having... When you look on Snapchat, most of the time, it just seems like you can see, like, the good things. Cos people filming it are like oh such good fun. They won't put on stuff that's like really bad. So it makes it; like I guess it gives the alcohol like a more positive view (ID 8b, F, non-drinker)*

2. *RES: I think whenever people are having like a night, like party then there's always like drinks on their Snapchat which just shows that they're having fun and they're drinking…*

   INT: Mm.

   RES: So I wanna do that. And go to more things like that. (ID 26, F, drinker)

For peer review only - http://bmjopen.bmj.com/site/about/guidelines.xhtml

S5. The pressurised environment

1. *INT: What kind of things would people say? What sort of um, type of thing would they be?*

RES: Just like – like calling them scared and stuff for not doing it and stuff like that.

INT: Okay. Yeah. Yeah.

RES: Like, are you too scared to do this? But like other words. (ID 33, M, drinker)

2. *.. a lot of people drink to impress their friends so say like one friends like oh yeah I can drink this much and then the others like well I can drink this much and it's almost like a sly competition and then that's when it gets bad and out of hand like I've seen that from friends and then they've lost friends like because of it. (ID 31a, F, moderate drinker)*

3. *RES: I think it's harder because what people want other people's – they want other people to think about them like in a more positive way like…*

INT: Hmm.

RES: … they don't wanna be thought of this person who's like not fun and doesn't want to do all the stuff… (ID 33, M, drinker)

4. *RES3: Some people think you have to, just like peer pressure, I guess, if you're at a party or something [laughs].*

RES2: Mm.

INT: What does that kind of feel like? What happens, when you say, 'peer pressure', what's...?

RES1: Forced into doing it, really. (ID 35a (M), b (F), c (F), non-drinkers)

For peer review only

5. *RES: I think they always teach like I think they always teach peer pressure wrong.*

INT: Right. So what do they say?

RES: I mean they - well they always just say like if someone asks you to do something that you don't want to do just say no. But it-it's not really that. I think it's more that people are doing things and if you are not part of it – well you're not – like if you're not doing it as well you're not part of it... (ID 7, F, non-drinker)

6. *RES: I don't think so. I think I haven't really ever experienced any, like, peer pressure. Um, but I still think there is quite a lot of pressure to drink, um, whether or not anyone means to pressurise you. (ID 32, F, drinker)*

Abbreviations:

INT: Interviewer

RES: respondent

For peer review only - http://bmjopen.bmj.com/site/about/guidelines.xhtml

COREQ (COnsolidated criteria for REporting Qualitative research) Checklist

A checklist of items that should be included in reports of qualitative research. You must report the page number in your manuscript where you consider each of the items listed in this checklist. If you have not included this information, either revise your manuscript accordingly before submitting or note N/A.

Topic	Item No.	Guide Questions/Description	Reported on Page No.
**Domain 1: Research team and reflexivity**			
Personal characteristics			
Interviewer/facilitator	1	Which author/s conducted the interview or focus group?	
Credentials	2	What were the researcher's credentials? E.g. PhD, MD	
Occupation	3	What was their occupation at the time of the study?	
Gender	4	Was the researcher male or female?	
Experience and training	5	What experience or training did the researcher have?	
Relationship with participants			
Relationship established	6	Was a relationship established prior to study commencement?	
Participant knowledge of the interviewer	7	What did the participants know about the researcher? e.g. personal goals, reasons for doing the research	
Interviewer characteristics	8	What characteristics were reported about the inter viewer/facilitator? e.g. Bias, assumptions, reasons and interests in the research topic	
**Domain 2: Study design**			
Theoretical framework			
Methodological orientation and Theory	9	What methodological orientation was stated to underpin the study? e.g. grounded theory, discourse analysis, ethnography, phenomenology, content analysis	
Participant selection			
Sampling	10	How were participants selected? e.g. purposive, convenience, consecutive, snowball	
Method of approach	11	How were participants approached? e.g. face-to-face, telephone, mail, email	
Sample size	12	How many participants were in the study?	
Non-participation	13	How many people refused to participate or dropped out? Reasons?	
Setting			
Setting of data collection	14	Where was the data collected? e.g. home, clinic, workplace	
Presence of non-participants	15	Was anyone else present besides the participants and researchers?	
Description of sample	16	What are the important characteristics of the sample? e.g. demographic data, date	
Data collection			
Interview guide	17	Were questions, prompts, guides provided by the authors? Was it pilot tested?	
Repeat interviews	18	Were repeat inter views carried out? If yes, how many?	
Audio/visual recording	19	Did the research use audio or visual recording to collect the data?	
Field notes	20	Were field notes made during and/or after the inter view or focus group?	
Duration	21	What was the duration of the inter views or focus group?	
Data saturation	22	Was data saturation discussed?	
Transcripts returned	23	Were transcripts returned to participants for comment and/or	

For peer review only

Topic	Item No.	Guide Questions/Description	Reported on Page No.
		correction?	
**Domain 3: analysis and findings**			
Data analysis			
Number of data coders	24	How many data coders coded the data?	
Description of the coding tree	25	Did authors provide a description of the coding tree?	
Derivation of themes	26	Were themes identified in advance or derived from the data?	
Software	27	What software, if applicable, was used to manage the data?	
Participant checking	28	Did participants provide feedback on the findings?	
Reporting			
Quotations presented	29	Were participant quotations presented to illustrate the themes/findings? Was each quotation identified? e.g. participant number	
Data and findings consistent	30	Was there consistency between the data presented and the findings?	
Clarity of major themes	31	Were major themes clearly presented in the findings?	
Clarity of minor themes	32	Is there a description of diverse cases or discussion of minor themes?	

Developed from: Tong A, Sainsbury P, Craig J. Consolidated criteria for reporting qualitative research (COREQ): a 32-item checklist for interviews and focus groups. *International Journal for Quality in Health Care*. 2007. Volume 19, Number 6: pp. 349 – 357

Once you have completed this checklist, please save a copy and upload it as part of your submission. DO NOT include this checklist as part of the main manuscript document. It must be uploaded as a separate file.

For peer review only
